# The *Caenorhabditis elegans* TDRD5/7-like protein, LOTR-1, interacts with the helicase ZNFX-1 to balance epigenetic signals in the germline

**DOI:** 10.1371/journal.pgen.1010245

**Published:** 2022-06-03

**Authors:** Elisabeth A. Marnik, Miguel V. Almeida, P. Giselle Cipriani, George Chung, Edoardo Caspani, Emil Karaulanov, Hin Hark Gan, John Zinno, Ida J. Isolehto, Fridolin Kielisch, Falk Butter, Catherine S. Sharp, Roisin M. Flanagan, Frederic X. Bonnet, Fabio Piano, René F. Ketting, Kristin C. Gunsalus, Dustin L. Updike

**Affiliations:** 1 The MDI Biological Laboratory, Bar Harbor, Maine, United States of America; 2 Husson University, Bangor, Maine, United States of America; 3 Institute of Molecular Biology, Mainz, Germany; 4 International PhD Programme on Gene Regulation, Epigenetics & Genome Stability, Mainz, Germany; 5 Center for Genomics & Systems Biology, New York University, New York, New York, United States of America; 6 Center for Genomics & Systems Biology, New York University Abu Dhabi, Abu Dhabi, United Arab Emirates; University of Cambridge, UNITED KINGDOM

## Abstract

LOTUS and Tudor domain containing proteins have critical roles in the germline. Proteins that contain these domains, such as Tejas/Tapas in *Drosophila*, help localize the Vasa helicase to the germ granules and facilitate piRNA-mediated transposon silencing. The homologous proteins in mammals, TDRD5 and TDRD7, are required during spermiogenesis. Until now, proteins containing both LOTUS and Tudor domains in *Caenorhabditis elegans* have remained elusive. Here we describe LOTR-1 (D1081.7), which derives its name from its **LO**TUS and **T**udo**r** domains. Interestingly, LOTR-1 docks next to P granules to colocalize with the broadly conserved Z-granule helicase, ZNFX-1. The Tudor domain of LOTR-1 is required for its Z-granule retention. Like *znfx-1* mutants, *lotr-1* mutants lose small RNAs from the 3’ ends of WAGO and mutator targets, reminiscent of the loss of piRNAs from the 3’ ends of piRNA precursor transcripts in mouse Tdrd5 mutants. Our work shows that LOTR-1 acts with ZNFX-1 to bring small RNA amplifying mechanisms towards the 3’ ends of its RNA templates.

## Author summary

Germ granules are protein and RNA complexes that are critical for maintaining an animal’s fertility. Central to the composition of germ granules are their small RNAs, which have the capacity to convey a memory of germline-licensed expression from one generation to the next. Here we describe and characterize a new germ-granule protein in *C*. *elegans* that we’ve named LOTR-1, after its LOTUS and Tudor domains. This combination of LOTUS and Tudor domains can be found in the mammalian proteins TDRD5 and TDRD7, which are required during spermatogenesis. During *C*. *elegans* embryogenesis, germ granules demix or partition into subgranules with refined functions. We show that LOTR-1 partitions with a specific class of subgranules called Z granules, interacting with a Z-granule helicase called ZNFX-1. Here, LOTR-1 functions with ZNFX-1 to position small RNA amplification from RNA templates, ensuring a memory of germline expression across generations. These findings may provide new insight into the function of TDRD5 and TDRD7 during human germline development.

## Introduction

Germ cells produce the next generation, and their pluripotent potential is instrumental for ensuring fertility and proper development. While germline and somatic cells contain the same DNA, differences within their cytoplasm, or germplasm, help distinguish their respective fates. [1] In some animals, ectopic germplasm can be sufficient to reprogram somatic nuclei. [2–5] Additionally, the presence of germ plasm components in the soma promotes cell proliferation, pluripotency, and tumorigenesis. [6–9] Understanding how the germplasm derives this reprogramming potential is a critical undertaking with far-reaching implications to reproductive, developmental, and regenerative biology.

The germplasm contains non-membrane-bound ribonucleoprotein condensates called germ granules that harbor part of this reprogramming potential. [10,11] One conserved feature of germ granules across species is the presence of proteins with LOTUS and Tudor domains. LOTUS is a name derived from the germ-granule-associated proteins **L**imkain/MARF1, **O**skar, and **Tu**dor-containing protein**s** 5 and 7. [12–15] The LOTUS domain takes on a winged helix-turn-helix (wHTH) folding pattern to facilitate RNA and protein interactions critical to germline development. *Drosophila* Oskar uses its LOTUS domain to self-dimerize and interface with the Vasa DEAD-box helicase, stimulating its activity to promote piRNA amplification in the germline. [16] Oskar expression in the *Drosophila* oocyte drives germ-granule assembly, while its mislocalization is sufficient to form ectopic germ cells from somatic progenitors. [17,18] However, drawing parallels between Oskar and other LOTUS-containing proteins is difficult as Oskar is confined to only some insects and likely arose *de novo via* a fusion of a eukaryotic LOTUS domain with a bacterially derived hydrolase-like domain called OSK through horizontal gene transfer. [19] In *Drosophila*, Limkain/MARF-1 regulates oocyte maturation and its LOTUS domain associates with the CCR4-NOT deadenylase complex. This results in the shortening of poly-A tails and translational regulation of targeted RNA transcripts. [20] In mice, MARF1 localizes to germ granules in oocytes, where it is critical for normal oocyte development. Female mice lacking MARF1 are sterile due to failures in oocyte meiosis and increased retrotransposon activity. [21,22] In addition to interfacing with proteins, LOTUS domains also bind RNA with a preference for G-rich RNAs and those that form a G-quadruplex (G4) secondary structure. [23] The function of these G-rich and G4 interactions remains unclear but could be instrumental to the role of LOTUS in RNA metabolism and regulation. [23]

TDRD5 and TDRD7 contain LOTUS domains paired with Tudor scaffolding domains. [24] Tudor domains have been shown to bind methylated arginines and lysines, with some preference for Argonaute proteins and histone tails. [25–27] In mammals, TDRD5 and TDRD7 associate with key components of the piRNA pathway and are required for normal spermatogenesis and retrotransposon silencing. [28–32] The TDRD5 and TDRD7 orthologs in *Drosophila*, respectively known as Tejas and Tapas, are required for proper germ granule formation and piRNA silencing of transposons in the germline. [33,34] Combined, these findings illustrate the importance of germ-granule localized LOTUS and Tudor domain-containing proteins in maintaining germline integrity through translational regulation, transposon silencing, and stimulation of Vasa helicase activity.

Germ granule studies are aided in *C*. *elegans* by the nematode’s transparency, permitting the observation of germ granules (or P granules) in living animals at all stages of development. During embryonic development, P granules segregate to germline blastomeres (P cells) before coming to reside in two primordial germ cells (PGCs). [35] Following the formation of PGCs, distinct sub-granules emerge from P granules to occupy neighboring sites at the nuclear periphery. [36] Known sub-granules include Z granules, SIMR foci, and Mutator foci—each containing sets of proteins that refine and resolve processes or steps of small RNA biosynthesis. [37–39]

*C*. *elegans* expresses several classes of small RNAs. [40,41] 21U-RNAs are considered the piRNAs of *C*. *elegans* due to their interaction with the germline-expressed PRG-1 Argonaute, the main Piwi-class Argonaute of *C*. *elegans*. [42–44] Similar to piRNAs of other organisms, PRG-1/21U-RNAs target “non-self” sequences such as transposable elements. [42,45,46] Target recognition leads to the recruitment of RNA-dependent RNA Polymerases (RdRPs), which synthesize complementary 22G-RNAs from template target RNAs. [46,47] In turn, 22G-RNAs associate with WAGO-class Argonautes, which elicit target gene silencing at the post-transcriptional and transcriptional levels. [47–51] An important function of PRG-1/21U-RNAs is to prevent the erroneous targeting of essential genes by 22G-RNAs. [52,53] Gene silencing initiated by 21U-RNAs can become independent of the initial PRG-1/21U-RNA trigger and self-sustained by 22G-RNAs and heterochromatin marks. [49,51,54] This PRG-1/21U-RNA-independent silencing is termed RNA-induced epigenetic silencing (RNAe). To prevent spurious silencing of endogenous genes, a distinct subpopulation of 22G-RNAs associates with CSR-1, the only essential Argonaute protein in *C*. *elegans*, to counteract PRG-1/21U-RNA complexes and license gene expression in the germline [55–57].

26G-RNAs are produced by the RdRP RRF-3 and additional cofactors in gonads. [58–63] Two distinct subpopulations of 26G-RNAs are expressed: those expressed during spermatogenesis that associate with the Argonautes ALG-3/4 [60,64,65], and those expressed during oogenesis and embryogenesis that associate with the Argonaute ERGO-1. [58,60,66] 26G-RNAs also trigger secondary 22G-RNA synthesis to produce robust target gene silencing. Due to their production downstream of several primary pathways, 22G-RNAs comprise a highly complex small RNA species. 22G-RNAs can be functionally divided into distinct subpopulations based on their expression pattern, the Argonaute protein with which they interact, and their set of target genes. [40,41]

In *C*. *elegans*, aspects of small RNA biogenesis and gene silencing occur both in the nucleus and cytoplasm. [40] Cytoplasmic reactions of small RNA pathways mostly take place in germ granules, as extrapolated by the localization of many small RNA cofactors to these granules. [67] The interplay of small RNA biogenesis and silencing seems complex, but the partitioning of germ granules into sub-compartments suggests that these processes are physically organized in a vectorial manner, similar to the multiphase liquid condensates that mediate ribosome biogenesis in the nucleolus. [68] For example, Mutator foci are considered the site of WAGO-class 22G-RNA biogenesis. [69] Z granules were initially defined by the localization of ZNFX-1, an RNA helicase required for inheritance of small RNAs and transgenerational germ cell homeostasis. [70] Interestingly, ZNFX-1 was shown to interact with Argonaute proteins and is required for the correct positioning of RdRPs in their target transcripts. [70,71] *znfx-1* mutants display unbalanced 22G-RNA synthesis, with higher 22G-RNA levels produced towards the 5’ of the target transcript. PID-2/4/5 are recently identified factors that affect the structure of Z granules, are required for germ cell immortality, and are similarly required to balance 22G-RNA signals. [38,39] These studies demonstrate a link between Z granules and the biogenesis and inheritance of 22G-RNAs, even though clearly defined molecular mechanisms that act in Z granules have not been identified.

The role of LOTUS-domain proteins in *C*. *elegans* has remained unexplored. However, three LOTUS containing proteins have recently been identified: MIP-1, MIP-2, and D1081.7. [72] We find that D1081.7 is in germ granules and interacts with both MIP-1 and MIP-2. D1081.7 is the only known *C*. *elegans* protein to pair LOTUS and Tudor domains, similar to both TDRD5 and TDRD7, so we have named it **LO**TUS and **T**udo**R** containing protein **1** (LOTR-1). The Tudor domain of LOTR-1 is required for its association with germ-granules, but its LOTUS domains are not. A quantitative mass spectrometry approach revealed a robust reciprocal association between LOTR-1 and ZNFX-1, while imaging showed that LOTR-1 partitions with ZNFX-1 into Z granules with the formation of PGCs, and that *lotr-1* mutants have smaller Z granules. Furthermore, LOTR-1, like ZNFX-1, is required for the normal distribution of PRG-1 and MIP-2, but only subtly impacts the localization of the germ-granule components GLH-1, DEPS-1, PGL-1, PGL-3, MIP-1 and ZNFX-1 in adult germ cells. *lotr-1* mutants, including one with an in-frame LOTUS deletion, have deregulated 22G- and 26G-RNAs and an altered distribution of WAGO/mutator class 22G-RNAs towards the 5’ end of some transcripts, similar to what has been described for *znfx-1* mutants.[71] In addition, these *lotr-*1 mutants have a mortal germline phenotype, and show enhanced inheritance of RNAi. We conclude that LOTR-1 functions with ZNFX-1 in Z granules to ensure balanced 22G-RNA biogenesis and the proper silencing of WAGO/mutator targets from one generation to the next. These findings provide new insights into Z granule composition and may help elucidate potential somatic and germline functions of TDRD5, TDRD7, and other proteins containing paired LOTUS and Tudor domains.

## Results

### LOTR-1 is a Z-granule protein that contains both LOTUS and Tudor domains

**Fig 1:**
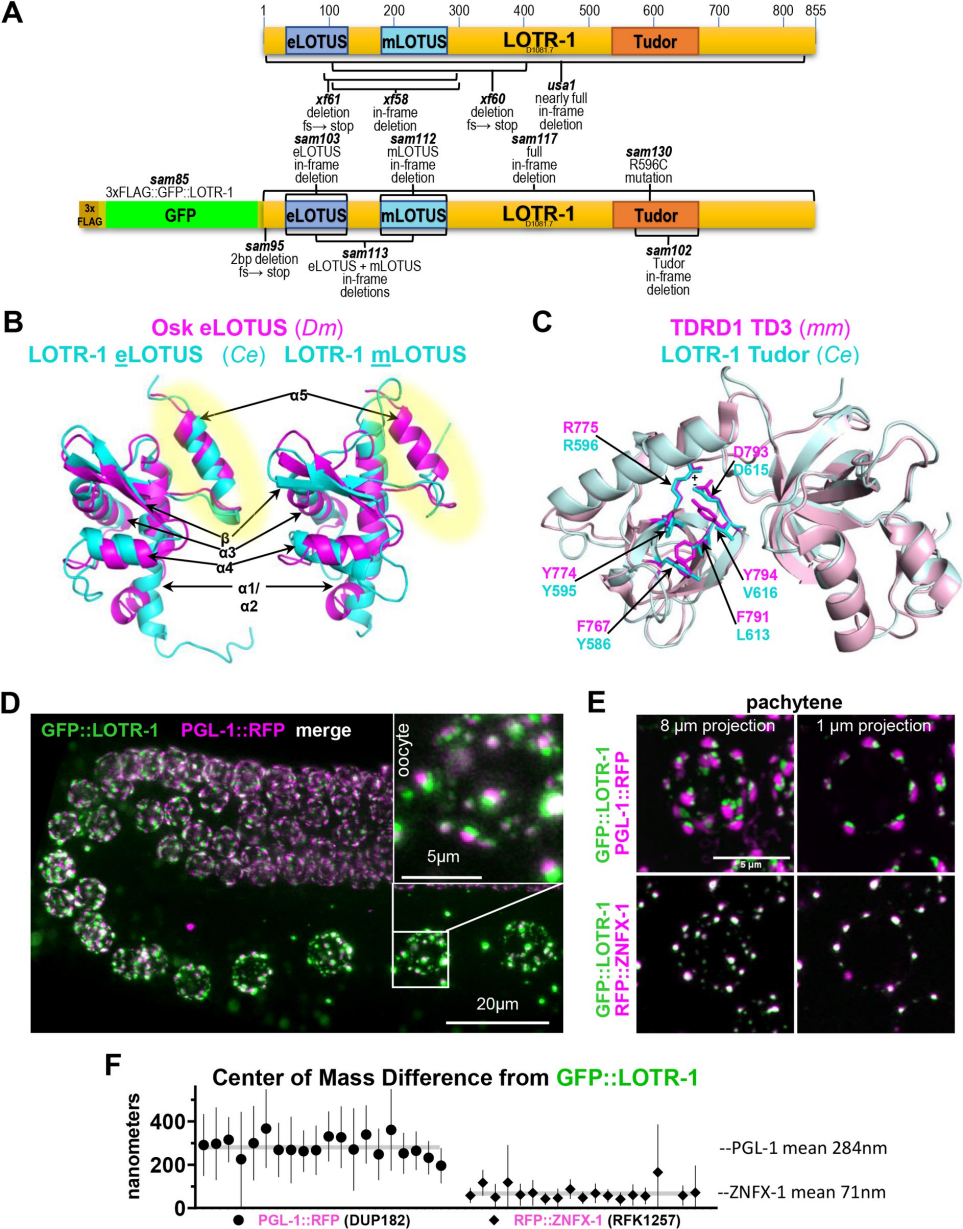
LOTR-1 contains both LOTUS and Tudor domains, and colocalizes with Z granules. A) Top schematic depicts the location of the eLOTUS, mLOTUS and Tudor domain of LOTR-1. Bottom depicts the CRISPR edit used to add an N terminal 3X FLAG tag and GFP. Alleles generated for this study are indicated. B) Predicted 3D model overlap of the eLOTUS and mLOTUS domains of *C*. *elegans* LOTR-1 with the Oskar eLOTUS domain from *Drosophila melanogaster*. The α5 helix (highlighted) is only present in the eLOTUS domain. C) Predicted 3D structure of the Tudor domain (aa 534–670) of LOTR-1 overlapped with TD3 domain of mouse TDRD1. D) GFP::LOTR-1 and PGL-1::RFP in the germline of living worms. E) Super-resolution confocal imaging of GFP::LOTR-1 with PGL-1::RFP (top) and GFP::LOTR-1 with RFP::ZNFX-1 (bottom) in pachytene germ cells. F) Comparison of the center of mass difference from LOTR-1 for PGL-1 or ZNFX-1.

Inaugural papers that first described the LOTUS winged-helix domain and its conservation identified *C*. *elegans* D1081.7 as a hypothetical orphan protein with a single LOTUS domain. [12,13] More recently, LOTUS domains have been subdivided into extended LOTUS (eLOTUS) domains (like the LOTUS domain in *Drosophila*’s Oskar), and minimal LOTUS (mLOTUS) domains that lack a conserved α5 helix (like those present in mammalian MARF1) (S1 Fig). [14] The LOTUS domain of D1081.7 described in these inaugural papers corresponds to the eLOTUS domain (aa 33–130), but our analysis uncovered an accompanying mLOTUS domain (aa 180–285) (Fig 1A). LOTUS domains have low sequence similarity and are challenging to identify using sequence analysis alone. The mLOTUS domain of D1081.7 shares sequence identity (21%) to the winged-helix region of Cdt1, a regulator in the DNA replication complex. [73] The predicted 3D structures of D1081.7 LOTUS domains superimpose well with solved structures from other species: eLOTUS domains of D1081.7 and Oskar align with a root-mean-square deviation (rmsd) of 2.8 Å, whereas mLOTUS, which lacks the α5 helix, deviates from both fly and human LOTUS folds by ~3.5 Å rmsd (Fig 1B). These paired LOTUS domains reflect the arrangement of TDRD5 and TDRD7 in mammals and two proteins recently described in *C*. *elegans* called MIP-1 and MIP-2 (S1 Fig). [72]

Default BLAST parameters fail to uncover the Tudor domain within D1081.7, but it readily appears in domain-enhanced searches and was previously identified using multiple sequence alignment. [12] The Tudor domain (aa 534–670) is most similar to Tudor domain 3 (TD3) of mouse TDRD1, which is known to bind symmetrically dimethylated arginine (sDMA) marks through a hydrophobic pocket that is created by the arrangement of four aromatic residues (Fig 1C, F767, Y774, F791, and Y794). [74] This pocket is stabilized by charge interactions between R775 and D793. The positions of these two residues are conserved in D1081.7 (R596 and D615). However, two of the four aromatic residues of the aromatic cage are replaced with other hydrophobic alternatives in D1081.7 (L613 and V616). How this impacts sDMA binding is unknown.

The combination of both LOTUS and Tudor domains in D1081.7 is unique to homologs of Tejas/TDRD5 and Tapas/TDRD7 in *Drosophila* and mammals, which are piRNA pathway-regulating components that localize to the perinuclear nuage of germ cells. D1081.7 is the only protein in *C*. *elegans* known to have both LOTUS and Tudor domains. The Tudor and LOTUS domains of TDRD7 and TDRD5 are arranged similarly to those of D1081.7, with TDRD5 sharing 34% sequence similarity to D1081.7 across 466 aa, and TDRD7 sharing 39% sequence similarity across 550 aa. While reciprocal BLASTP searches suggest that D1081.7 homologs are confined to class V nematodes (S1A), its domain architecture and predicted structure suggests that D1081.7 is a putative homolog of TDRD5 and TDRD7 in *C*. *elegans* (S1D). Given these structural features, D1081.7 was named LOTUS and Tudor domain protein 1 (LOTR-1).

To determine the expression of LOTR-1, CRISPR/Cas9 genome editing was used to place an N-terminal GFP tag on endogenous *lotr-1* in a strain carrying PGL-1::RFP. LOTR-1 localized to germ granules throughout the adult germline; however, LOTR-1 granules appeared docked next to P granules marked by PGL-1::RFP (Fig 1D). Super-resolution confocal imaging of pachytene germ cells confirmed that LOTR-1 and PGL-1 granules appear adjacent to one another in the adult germline (Fig 1E, top), with their center of mass separated by an average of 284nm (Fig 1F). This pattern is similar to the P-granule docking of Z granules, Mutator foci, and SIMR foci, suggesting that LOTR-1 could reside within these germ granule sub-compartments. [70,71,75] To test this, LOTR-1 was examined for colocalization with RFP::ZNFX-1, which defines Z granules. Red and green channels show significant overlap (Fig 1E, bottom), with their center of mass separated by an average of only 71nm (Fig 1F). These results suggest that LOTR-1 partitions with Z granules instead of P granules in the adult germline.

### Functional analysis of LOTR-1 domains reveals effects on subcellular localization and fertility

**Fig 2:**
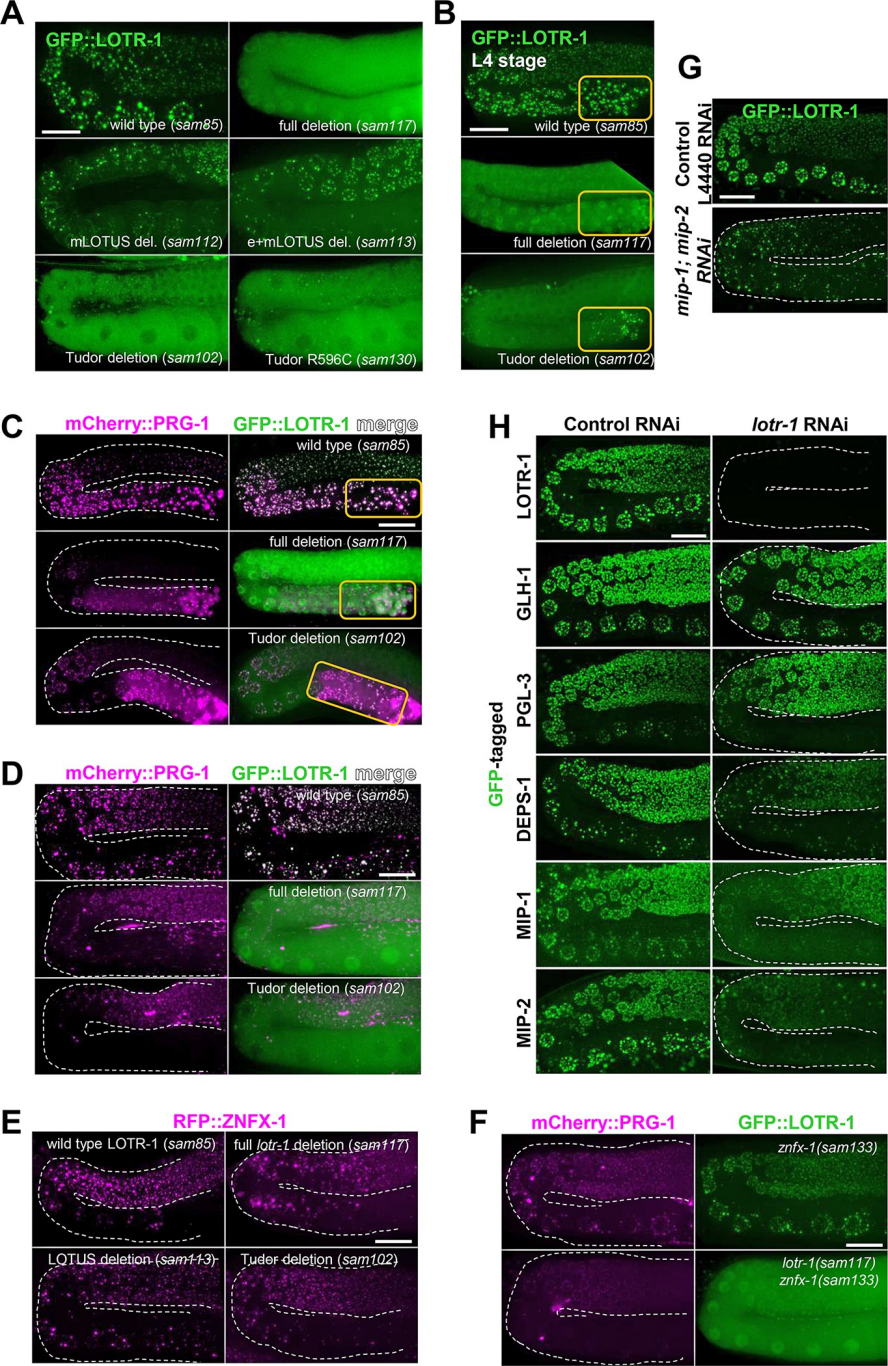
Germ-granule phenotypes in *lotr-1* germlines. A) Live-imaging of young adults shows the distribution of LOTR-1::GFP in the presence and absence of its LOTUS and Tudor domains. B) LOTR-1 distribution during spermatogenesis in the fourth larval stage (L4). Orange boxes indicate region of spermatogenesis. Live imaging of mCherry::PRG-1 and GFP::LOTR-1 in C) L4 stage and D) young adult germlines. E) Live imaging of RFP::ZNFX-1 in the germline of wild-type and *lotr-1* mutant adults. F) Comparison of GFP::LOTR-1 and mCherry::PRG-1 expression in the germlines in *znfx*-1 mutant (top) and in *lotr-1; znfx-1* double mutant (bottom) adults. G) RNAi depletion of *mip-1* and *mip-2* in GFP::LOTR-1 compared to empty vector control RNAi. H) *lotr-1* RNAi compared to empty vector control RNAi in adult germlines of GFP-tagged LOTR-1, GLH-1, PGL-3, DEPS-1, MIP-1 and MIP-2 worms. Scale is 20 microns. Quantification provided in S2 Fig.

To understand how LOTR-1 is recruited to germ granules, several point mutations and deletions were introduced into the 3xFLAG::GFP::LOTR-1 strain (Fig 1A). Deletions of the mLOTUS and combined eLOTUS/mLOTUS domains do little to disperse truncated LOTR-1 from germ granules in young adults (Fig 2A) or reduce LOTR-1 granule number and size (S2A Fig). In contrast, the Tudor domain deletion disperses LOTR-1, decreasing LOTR-1 granule size and reducing the number of LOTR-1 granules in the adult germline by 85% (Figs 2A and S2A). A point mutation in the conserved arginine (R596C) of LOTR-1, which is predicted to stabilize the pocket with the potential to bind sDMAs, disperses LOTR-1 as well (Figs 2A and S2A).[37] These results suggest that the germ-granule association of LOTR-1 depends primarily on its Tudor domain, potentially mediated through well-characterized Tudor/sDMA interactions. In the absence of the Tudor domain, 37% of LOTR-1 granules are still retained during spermatogenesis in the fourth larval stage (Fig 2B, orange box, S2B Fig), suggesting that its retention in spermatogenic germ granules is not fully dependent on Tudor/sDMA interactions. One possibility is that when the nuclear envelope breaks down during spermatogenesis, causing germ granules to condense into fewer and larger aggregates, these larger granules facilitate LOTR-1 association even without its Tudor domain. Interestingly, *lotr-1* mutants show only a subtle reduction in brood size in comparison to wild type, even after a generation grown at 26° C, in contrast to the robust temperature-sensitive sterile phenotypes of other P-granule mutants, suggesting a more specialized role for LOTR-1 in neighboring Z granules (S2C Fig).

Tudor domains interact with PIWI Argonaute proteins via sDMAs. [76] To see if this interaction extends to *C*. *elegans* Argonautes, the distribution of the PIWI homolog PRG-1 was examined in the presence and absence of LOTR-1, and in a strain carrying LOTR-1 with the Tudor domain deletion. Under wild-type conditions, PRG-1 is constitutively associated with germ granules from the distal germline through gametogenesis, with more abundant expression during spermatogenesis. In both the *lotr-*1 full- and Tudor deletion backgrounds, 60–72% of PRG-1 granules persist during spermatogenesis, respectively (Fig 2C, orange box, S2B Fig). Only 29–42% of PRG-1 granules persist in pachytene germ cells, and 6%-28% in oocytes, in indicated *lotr-1* mutant adults (Figs 2D and S2D and S2E). Both LOTR-1 and PRG-1 are deposited in residual bodies during spermatogenesis and absent from spermatids in the presence or absence of the Tudor domain of LOTR-1 (S2F Fig). These findings suggest that LOTR-1’s Tudor domain is not necessary to localize PRG-1 to germ granules, but the Tudor domain enhances this localization, potentially through interactions with sDMAs, more in oocytes than during spermatogenesis.

The impact of LOTR-1 is less pronounced on ZNFX-1 than it is on PRG-1 localization. In *lotr-1* (deletion) germlines, 83% of ZNFX-1 granules persist in pachytene cells, and 61% persist in oocytes (Figs 2E and S2G and S2H). There was no significant difference in the number of ZNFX-1 granules in the *lotr-1* LOTUS and Tudor deletion backgrounds, suggesting that the presence of LOTR-1 is not required for ZNFX-1 localization. An early frameshift mutation was introduced into ZNFX-1 to determine whether loss of ZNFX-1 reciprocally impacts GFP::LOTR-1 and RFP::PRG-1 expression. LOTR-1 localization remains largely intact in the *znfx-1* mutant (Fig 2F). While PRG-1 localization looks wild-type in the first generation of *znfx-1* homozygotes, only 66% (pachytene) and 54% (oocyte) of PRG-1 granules persist in subsequent generations (Figs 2F and S2D, S2E and S2 I). This is reduced to 32% (pachytene) and 16% (oocyte) in the *lotr-1; znfx-1* double mutant, similar to levels in observed in *lotr-1* single mutant, suggesting that the ability of *lotr-1* and *znfx-1* to disperse PRG-1 is not additive.

Next, LOTR-1 localization was examined in the absence of two other recently characterized LOTUS-containing proteins, MIP-1 and MIP-2. [72] MIP-1 and MIP-2 are MEG-3 interacting proteins that localize to P granules throughout development and promote germ granule condensation. Although MIP-1 and MIP-2 lack Tudor domains, they each contain two eLOTUS domains that could indicate some functional synergy with LOTR-1 (S1D Fig). RNAi depletion of *mip-1* and *mip-2* together has been shown to cause the dispersal of PGL-1, PGL-3, and GLH-1. [72] Similarly, GFP-tagged LOTR-1 granule numbers are reduced 41% and are less prominent around the nuclear periphery following *mip-1*; *mip-2* RNAi, even though LOTR-1 is associated with Z and not P granules at this stage (Figs 2G and S2J). These results indicate that the LOTUS-domain proteins MIP-1 and MIP-2 directly or indirectly affect the localization of LOTR-1 at germ granules. GFP::LOTR-1 levels do not decrease with *mip-1; mip-2* RNAi, suggesting that LOTR-1 granule loss is attributed to relocation and not depletion (S3H Fig). In contrast, MIP-1 and MIP-2 are not significantly dispersed from pachytene germ granules in *lotr-1* mutants (S2J Fig). We sought to address whether LOTR-1 is required for localization of other germ-granule factors. GLH-1, PGL-3, DEPS-1 and MIP-1 GFP granules showed subtle, but non-significant, dispersal in the adult germline with *lotr-1* RNAi, while MIP-2 granules were dispersed by 60% with *lotr-1* RNAi, despite a slight increase in average MIP-2::GFP intensity (Figs 2H and S2K, S2L and S3H). This, together with the reciprocal effect of MIPs on LOTR-1 localization, suggests potential synergy between these three LOTUS-domain containing proteins.

### LOTR-1 binding partners include ZNFX-1, germ-granule proteins, and the cytoskeleton

To explore the potential for direct associations between LOTR-1, GLH-1, MIP-1, MIP-2, and ZNFX-1 we performed yeast-two-hybrid (Y2H) analyses. MIP-1 and MIP-2 have previously been shown to both homo- and heterodimerize, as well as associate with GLH-1/Vasa through their N-terminal LOTUS domains. [72] Y2H confirmed associations between LOTR-1 and both MIP-1 and MIP-2 (S3A Fig), whereas no interaction between LOTR-1 and GLH-1 was detected (S3B Fig). Surprisingly, the association of MIP-1 and MIP-2 with LOTR-1 appears to be mediated through the C-terminal ends of these proteins and not through their LOTUS domains (S3A Fig). The Y2H also suggests that both MIP-1 and MIP-2 interact more strongly with LOTR-1 when one or both of its two LOTUS domains are removed (S3A Fig). Although Y2H does not show an interaction between LOTR-1 and the GLH-1 helicase, binding was confirmed between LOTR-1 and the ZNFX-1 helicase (S3C Fig). This suggests that distinct helicase/LOTUS-domain-protein pairs (GLH-1:MIP-1/2 and ZNFX-1:LOTR-1) exist in neighboring P and Z granules.

**Fig 3:**
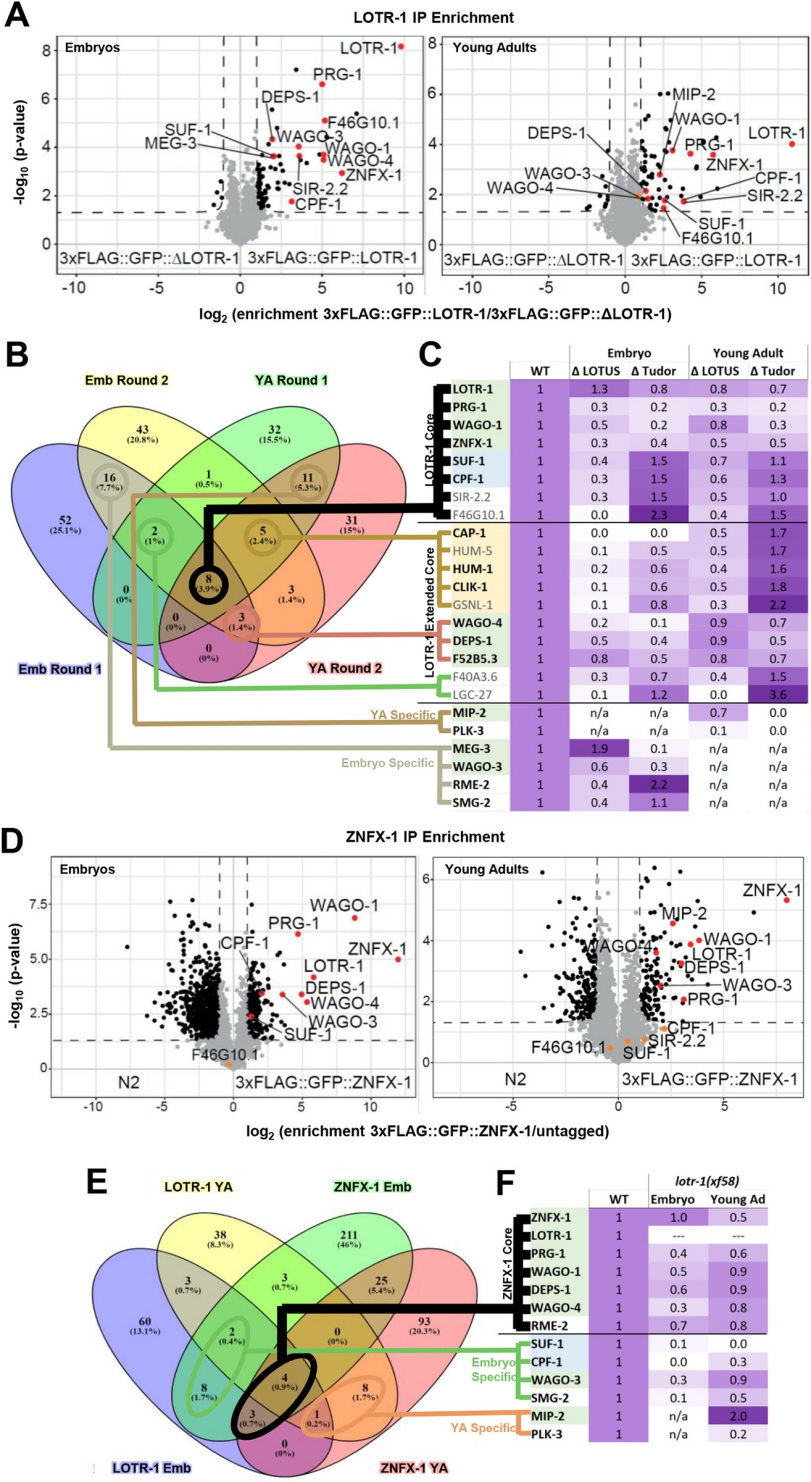
Quantitative IP-mass spectrometry analysis of LOTR-1 and ZNFX-1 immunoprecipitations. A) Volcano plots show the significance and enrichment of proteins that immunoprecipitated with 3xFLAG::GFP::LOTR-1 over the *lotr-1* deletion expressing GFP::3xFLAG alone, as identified by qMS. B) Venn diagram shows significantly enriched proteins that overlapped between two rounds of LOTR-1 IP-qMS from both embryo and young adult lysates. C) Table showing the change in LOTR-1 association in embryos and young adults when LOTUS or Tudor domains of LOTR-1 were deleted. D) Volcano plots show the significance and enrichment of proteins immunoprecipitated with 3xFLAG::GFP::ZNFX-1 over untagged ZNFX-1, as identified by qMS. E) Venn diagram shows significantly enriched proteins that overlapped between LOTR-1 and ZNFX-1 IP-qMS from both embryo and young adult lysates. F) Table showing the change in ZNFX-1 association in embryos and young adults when *lotr-1* was mutated. For C and F, a purple gradient was applied to visualize fold increases (>1, darker) and decreases (<1, lighter) from the C) LOTR-1 and F) ZNFX-1 associations identified in the wild type (WT) replicates.

To complement the Y2H analysis, we took an unbiased, biochemical approach to find additional LOTR-1 interactions. The N-terminal 3xFLAG tag introduced into the endogenous LOTR-1 locus was used to immunoprecipitate LOTR-1 from both young adults and embryos for quantitative mass spectrometry (IP-qMS) (S3D Fig). Two independent anti-FLAG IP-qMS experiments were performed, both in young adults and embryos, which allowed us to identify proteins differentially enriched in 3xFLAG::GFP::LOTR-1 over 3xFLAG::GFP::*lotr-1(deletion)* IPs for both young adults and embryos (Fig 3A and 3B and S1 Table). Eight proteins, including LOTR-1, were enriched in all four LOTR-1 IP datasets, two embryo datasets and two young adult datasets (Fig 3A–3C). After LOTR-1, the most highly enriched among these was the ZNFX-1 helicase. The other six proteins are the germ granule-associated Argonautes PRG-1 and WAGO-1, the 3’UTR cleavage and stimulation factors SUF-1 and CPF-1, the histone deacetylase SIR-2.2, and F46G10.1. These eight proteins represent a central core of possible LOTR-1 interactions, reinforcing the role of LOTR-1 in germ granules and small RNA biogenesis.

Expanding the interaction list by an additional ten proteins that showed significant enrichment in at least three out of the four embryo and young adult datasets, now includes the germ-granule proteins DEPS-1, the WAGO-4 Argonaute, and the YHTDC2-like DExH-helicase (F52B5.3) that was previously shown to interact with the germ-granule Argonaute CSR-1 (Fig 3B and 3C). [77] Of these 18 LOTR-1-interacting proteins, 5 are known to bind actin and regulate the cytoskeleton, including CAP-1, HUM-1, HUM-5, CLIK-1, and GSNL-1. [78–81] 12 of the 18 proteins originate from transcripts abundantly expressed in the germline (Fig 3C, bold), while transcripts encoding the remaining six (HUM-5, GSNL-1, F40A3.6, SIR-2.2, F46G10.1, and LGC-27) are lowly expressed in the germline and may reflect LOTR-1 interactions in whole-worm or embryo lysates that are not replicated *in vivo*. [82,83]

To distinguish if LOTR-1 associations obtained by co-immunoprecipitation (co-IP) are facilitated through its LOTUS or Tudor domains, LOTR-1 carrying deletions in either the e+mLOTUS (*sam113*) or Tudor (*sam102*) domains were immunoprecipitated from embryos and young adults and then analyzed with qMS to look for increased (>1, Fig 3C) or decreased (<1, Fig 3C) interactions. Anti-FLAG westerns confirmed expression of tagged LOTR-1 constructs at wild-type levels or above (S3E Fig). Deleting the LOTUS domains had a more pronounced effect than deleting the Tudor domain on the association of LOTR-1 with cytoskeletal proteins (reduced 80–100% in embryos, 50–70% in adults, Fig 3C, orange), 3’ UTR-cleavage and stimulation factors (reduced 60–70% in embryos, 30–50% in adults, Fig 3C, blue). LOTR-1 associations with other germ-granule proteins were decreased with both LOTUS and Tudor deletions (embryos and adults, Fig 3C, green). The effect was slightly more pronounced in the absence of the Tudor domain, which disperses LOTR-1 from germ granules. RFP::ZNFX-1 intensity levels are not significantly changed in *lotr-1* mutants (S3H Fig). This suggests that the weakened interaction with LOTR-1 proteins bearing LOTUS or Tudor domain deletions is due to the absence of these domains, and not to altered ZNFX-1 protein levels in *lotr-1* mutants.

Of the LOTR-1 associations specific to both embryo but not adult IPs, MEG-3 and WAGO-3 stand out because of their previously described association with germ granules. The association of LOTR-1 with MEG-3 in embryos is reduced 90% in the Tudor deletion, which shows this interaction is dependent on the Tudor domain of LOTR-1 (Fig 3C and S1 Table). Interestingly, the interaction with MEG-3 is enhanced almost two-fold in the absence of the LOTUS domains of LOTR-1. This may suggest that Tudor-dependent interactions between MEG-3 and LOTR-1 during embryogenesis are kept in check by other associations mediated through the LOTUS domains of LOTR-1. In turn, some LOTR-1 associations are more prominent in young adults. For example, MIP-2 associates with LOTR-1 in the young adult germline and this interaction primarily relies on the Tudor domain (Fig 3C and S1 Table). This is consistent with our Y2H results showing that MIP-2 interacts with the Tudor-domain-containing C-terminus of LOTR-1 (S3A Fig). Another of the more pronounced LOTR-1 interactors specific to young adults is the polo-like kinase PLK-3, and this interaction is disrupted when either the LOTUS or Tudor domains of LOTR-1 are removed (Fig 3C). The significance of these embryo and young adult-specific interactions warrants additional attention.

To confirm whether ZNFX-1 interactions reflect the core set of proteins bound to LOTR-1, anti-FLAG IP-qMS was performed on embryos and young adults expressing 3xFLAG::GFP::ZNFX-1 or an untagged control (Figs 3D and S3G). In addition to ZNFX-1, proteins enriched over untagged control in both embryo and young adult included LOTR-1 and its interactors (i.e., PRG-1, DEPS-1, WAGO-1, WAGO-4 and RME-2) (Fig 3D–3F). SMG-2, WAGO-3, and the 3’ UTR cleavage and polyadenylation factors SUF-1 and CPF-1 were enriched in embryos only, while MIP-2 and PLK-3 were again only enriched in young adults (Fig 3E and 3F). These results support a substantial overlap of interactions between ZNFX-1 and LOTR-1 within Z granules. To determine which of these ZNFX-1 associations depend on LOTR-1, ZNFX-1 IP-qMS was performed in both embryo and adult *lotr-1(xf58)* mutants, which harbor an in-frame deletion that removes most of the LOTUS domain (Figs 3F and 1A). The association of ZNFX-1 with PRG-1, DEPS-1, WAGO-1, WAGO-3 and WAGO-4 is reduced 40–70% in the *lotr-1* mutant embryos, while its association with SUF-1, CPF-1, and SMG-2 in embryos is practically eliminated (Fig 3E and 3F and S2 Table). This suggests that LOTR-1 stabilizes ZNFX-1 interactions with Argonaute proteins while acting as the link between ZNFX-1 and mRNA/3’UTR binding factors. Interestingly, the LOTUS-containing protein MIP-2 increases its association with ZNFX-1 two-fold in *lotr-1* mutant adults, which may support a compensatory function for MIP-2 and LOTR-1.

### *lotr-1* mutants deregulate subsets of 26G- and 22G-RNAs and display changes in 22G-RNA coverage

Having established that LOTR-1 interacts with ZNFX-1 in Z granules, we sought to compare mutant phenotypes. Mutations affecting small RNA biogenesis and amplification frequently exhibit transgenerational sterility or mortal germline (Mrt) phenotypes. [40,84] To address if LOTR-1 is required for transgenerational germline health, fertile generations were counted until sterility ensued for three different *lotr-1* mutant alleles (S4A Fig). Each of three *lotr-1* alleles failed to propagate beyond 50 generations, while wild-type (N2) worms remained fertile. Because *znfx-1* mutants have transgenerational epigenetic inheritance defects that manifest after several generations at 25° C [70], brood sizes of single and double *lotr-1; znfx-1* mutants were compared to wild-type broods over the course of five generations (S4B Fig). While brood sizes are similarly and significantly decreased in both *lotr-1* and *znfx-1* mutants by the fifth generation, the effect was not additive, again suggesting that LOTR-1 and ZNFX-1 act in the same pathway.

The Mrt phenotype and impact on germ granules observed in *lotr-1* suggest these mutants are defective in some aspect of small RNA biogenesis. Since many LOTUS domain proteins have functions in piRNA biology, [76] we asked if this is the case for LOTR-1 using lines that carry a 21U-RNA/piRNA sensor construct. This transgene contains a reporter for GFP::H2B expression that has been silenced through a piRNA target site in its 3’UTR; mutations affecting piRNA biogenesis or secondary 22G-RNA production de-silence expression of this transgene. [45] Loss-of-function alleles of *lotr-1* were crossed into two different sensor lines carrying this transgene: one in which silencing is still dependent on 21U-RNAs (S4C Fig), and another under stable RNAe that is maintained independent of the initial 21U-RNA trigger (S4D Fig). Mutations in *lotr-1* were unable to activate either piRNA sensor strain (S4C and S4D Fig), showing that LOTR-1 is not required for 21U-RNA-dependent silencing or for RNAe.

**Fig 4:**
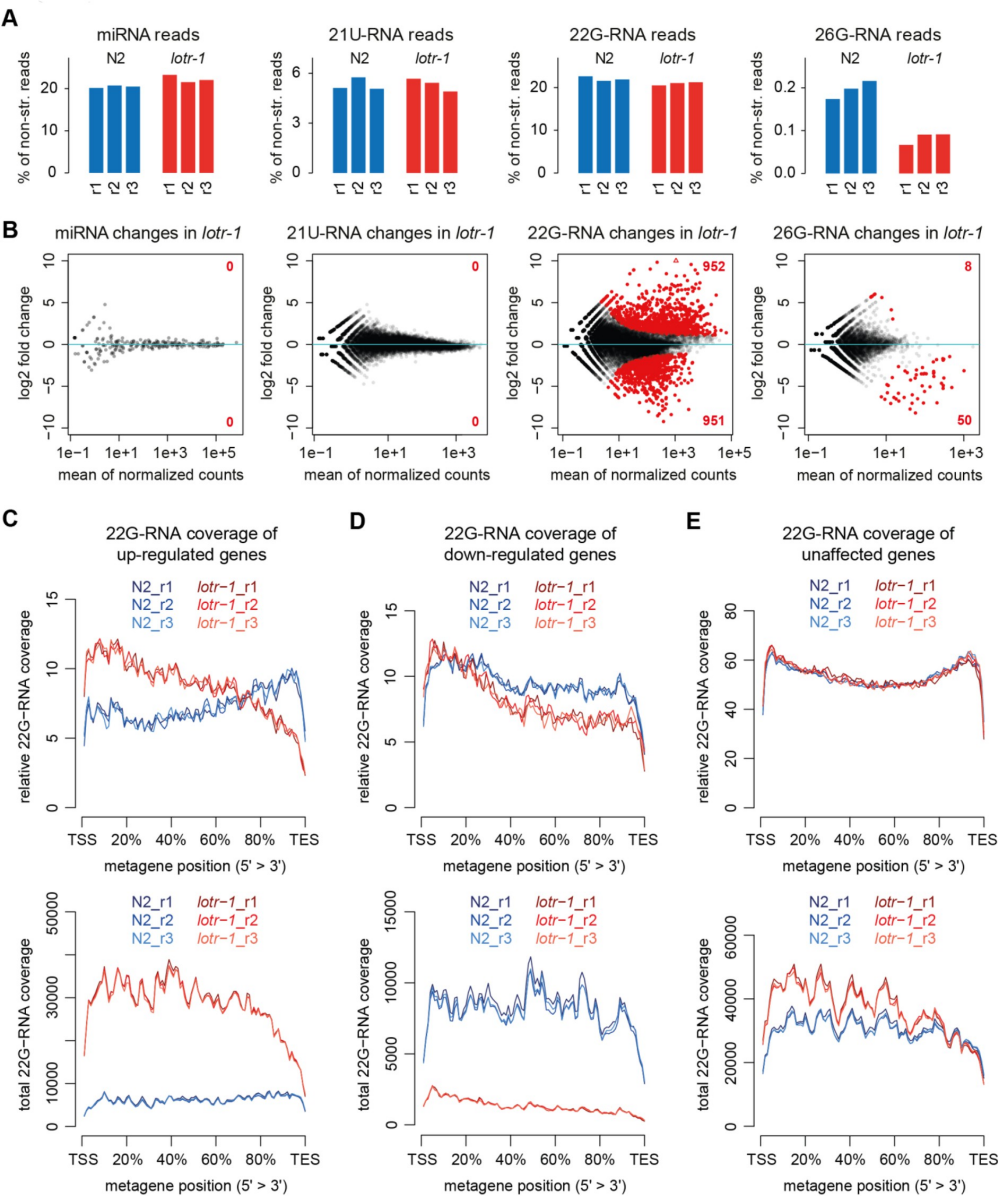
26G- and 22G-RNA deregulation in *lotr-1* mutants. A) Relative levels of the indicated small RNA populations normalized to all non-structural reads. B) Differential expression analysis to determine genes and transposons that are significantly depleted or enriched of mapped small RNAs in *lotr-1* mutants. The MA plots depict DESeq2 differential results for miRNAs (n = 257), 21U-RNAs over 21ur loci (n = 14328), and 22G-/26G-RNAs over protein coding genes (n = 20222), lincRNAs (n = 172), pseudogenes (n = 1791) and transposons (n = 151); significant changes (>2-fold at 10% FDR) are colored in red with the number of up- and down-regulated hits indicated. C-E) Metagene plots depicting relative (normalized per individual gene as in [71], upper panels) as well as total (lower panels) 22G-RNA coverage over genes whose 22G-RNAs are up-regulated, down-regulated or unaffected in the *lotr-1* mutants. Note that the total (cumulative) coverage plots utilize DESeq2-based normalization for library composition and are biased towards genes with strong 22G-RNA levels. TSS/TES, transcription start/end site.

As described in the introduction, *C*. *elegans* expresses a variety of small RNA species. [40] To address if LOTR-1 is required for some aspect of small RNA biogenesis, we sequenced small RNAs in wild-type and in *lotr-1(sam117)* (with the entire *lotr-1* coding sequence deleted, see Fig 1A). Small RNA sequencing in gravid adults showed that levels of 21U-RNAs and miRNAs are not significantly changed in *lotr-1* mutants (Fig 4A and 4B). The 26G-RNA pool is significantly affected and fifty genes show depletion of 26G-RNAs (Fig 4A and 4B). Of these, all but two are annotated to be targets of the known effectors ERGO-1, NRDE-3, or ALG-3/4, but the majority are ERGO-1 targets (47/50, S3 Table). [63,66,85] A GFP::NRDE-3 transgene, which localizes from the nucleus to the cytoplasm upon disruption of the 26G-RNA pathway, [63,86] remained expressed in the nucleus when crossed into *lotr-1* mutants (S4F Fig), indicating the 26G-RNA pathway is not critically impaired. It has been recently reported that deregulation of mutator-dependent 22G-RNAs targeting the *eri-6/7* locus may destabilize ERI-6/7, a protein required for ERGO-1-dependent 22G-RNA biogenesis [87]. In l*otr-1* mutants, we find a mild increase of 22G-RNAs in this locus (S4G Fig), which may explain the lower levels of ERGO-1-dependent 26G-RNAs (Fig 4A). Taken together, these results suggest that while LOTR-1 is not absolutely required for normal 26G-RNA biogenesis and silencing, a subset of 26G-RNA targets is affected by the depletion of LOTR-1.

Global 22G-RNA levels are not changed overall; however, hundreds of genes have deregulated 22G-RNA levels in *lotr-1* mutants (Fig 4A and 4B and S3 Table). Next, we asked if the deregulated 22G-RNAs map to particular gene classes. In *lotr-1* mutants, the majority of genes with deregulated 22G-RNAs are protein-coding genes (S5A Fig). 67 transposons display significantly up-regulated 22G-RNAs (S5A Fig and S3 Table), e.g. aggregated data for Tc1 transposons show approximately 3-fold increased 22G-RNA levels in *lotr-1* mutants (FDR-adjusted p-value of 2.13E-95, S3 Table). However, a reporter for Tc1 transposon mobility was not affected by the *lotr-1* mutation, suggesting that the upregulation of Tc1 22G-RNAs observed in *lotr-1* animals does not disrupt Tc1 silencing (S4E Fig). After separating 22G-RNAs into subgroups according to their Argonaute cofactors and target genes [40], we find that most genes with deregulated 22G-RNA levels in *lotr-1* mutants belong to the mutator and/or WAGO classes (S5B Fig and S3 Table). Overall, these results suggest that hundreds of genes show deregulated 22G-RNA levels, both increased and decreased, in *lotr-1* mutant animals across a range of gene classes and 22G-RNA targets.

In *znfx-1* mutants, 22G-RNA coverage is altered, revealing a shift toward the 5’ end of the target RNAs. [71] Metagene analysis of 22G-RNA coverage along transcripts showed a similar effect in the genes with deregulated 22G-RNA levels in *lotr-1* mutants, with stronger 5’ shift in the up-regulated genes (Figs 4C–4E and S5C and S5D). WAGO and mutator targets showed a similar 5’ shift of 22G-RNAs in *lotr-1* mutants compared to wild-type (S5C–S5F Fig). This 5’ shift of 22G-RNAs is not observed across all loci with robust 22G-RNA targeting (S5G Fig). Unlike *znfx-1*, the 5’ shift in 22G-RNA coverage in *lotr-1* mutants is observed in WAGO/mutator, but not in CSR-1 targets (S5H Fig). Notably, CSR-1 was not enriched in LOTR-1 IP-qMS, in either the embryo or young adult lysates. 22G-RNA coverage of ALG-3/4 targets is affected mainly in the 5’ region, whereas ERGO-1 targets are not affected in *lotr-1* mutants (S5I–S5J Fig). Of note, similar 22G-RNA deregulation and changes in 22G-RNA distribution across WAGO/mutator targets are also observed upon sequencing small RNAs from other *lotr-1* mutant alleles with partial deletions in the coding sequence (S5K–S5M Fig). Therefore, we conclude that LOTR-1 functions with ZNFX-1 to balance the production of 22G-RNAs across WAGO/mutator, but not CSR-1, target RNAs.

### LOTR-1 affects the inheritance of RNAi

**Fig 5:**
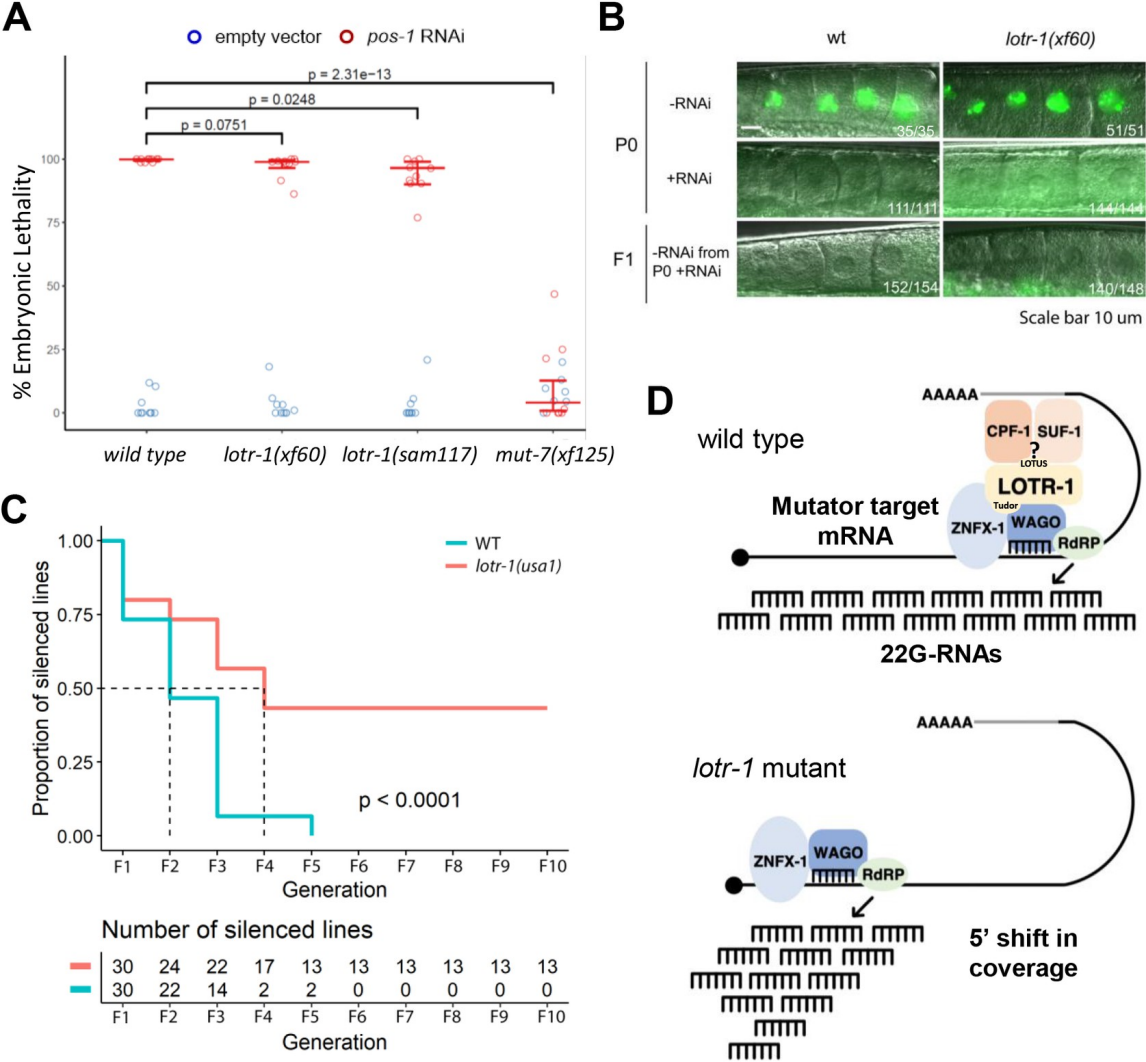
RNAi sensitivity and inheritance in *lotr-1* mutants. A) Sensitivity of two different *lotr-1* mutant alleles against *pos-1* RNAi, using *mut-7(xf125)* mutants as a known *pos-1* RNAi resistant control. Hatching proportion (y-axis) was scored; horizontal blue lines represent the fitted hatching probabilities. P-values were determined using Likelihood Ratio Tests. Error bars: bootstrap 95% confidence intervals for hatching probabilities. B) Inheritance of RNAi was tested using RNAi against GFP, targeting a transgene expressing a GFP::histone2B fusion (*mjIS31*) in oocytes [50]. Silencing in the treated P0 generation was fully effective, as was the inheritance of silencing to the F1. C) Transgenerational inheritance assay using RNAi against a GFP::histone2B transgene *(ruIs32)* in WT and the deletion allele *lotr-1(usa1*) revealed enhanced silencing up to 10 generations in the absence of LOTR-1. D) A model summarizing how LOTR-1, together with ZNFX-1, could affect 22G RNA production from an RdRP-targeted transcript. We note that all interactions are based on IP-qMS and Y2H experiments. Since these techniques do not report on whether a given interaction reflects a stable complex, or rather a more transient type of interaction, further study will be required to define the precise composition and nature of the associated molecular machinery.

The deregulation of 22G-RNA coverage over WAGO/mutator targets observed in *lotr-1* mutants, and the interaction of LOTR-1 with ZNFX-1 and WAGO proteins, may point to defects in exogenous RNAi and its inheritance. To test whether this is the case, the incidence of embryonic lethality following *pos-1* RNAi was examined in wild type and two *lotr-1* alleles, using a *mut-7* mutant as a control known to be resistant against *pos-1* RNAi (Fig 5A). Embryonic lethality was mildly suppressed in the full deletion allele, but not to the level observed in *mut-7* (Fig 5A, left). Next, we tested for defects in the inheritance of RNAi, using RNAi against GFP in wild type and *lotr-1* mutants carrying an H2B::GFP reporter. GFP RNAi was 100% effective in knocking down GFP expression in both wild-type and *lotr-1* worms. In contrast to *znfx-1* mutants [70], *lotr-1* mutants faithfully inherited the GFP silencing to the F1 generation (Fig 5B). In a separate experiment, inheritance of H2B::GFP silencing in *lotr-1* deletion mutants was followed for ten generations in isolated lineages, which revealed that *lotr-1* mutants can display enhanced RNAi inheritance (Fig 5C). Thus, although LOTR-1 and ZNFX-1 interact and the respective mutants share phenotypes, they clearly have distinct functions.

## Discussion

Considering the central role of *Drosophila* Oskar and other LOTUS domain-containing proteins like Tejas/TDRD5 and Tapas/TDRD7 in germline specification and development, we sought to ask whether LOTUS domain proteins in *C*. *elegans* function similarly and could thus be used to model TDRD5 and TDRD7 function in mammals. We have shown that LOTR-1 is the only protein in *C*. *elegans* where extended and minimal LOTUS domains are paired with a C-terminal Tudor domain, suggesting homology with TDRD5 and TDRD7 (S1 Fig).

ZNFX-1 and LOTR-1 colocalize within Z granules (Fig 1), have overlapping binding partners (Fig 3), balance production of 22G-RNAs across their targets (Fig 5), and *lotr-1; znfx-1* double mutants have a similar impact on PRG-1 dispersal as *lotr-1* single mutants (Figs 2 and S2). However, differences in the interaction partners of ZNFX-1 and LOTR-1 point to distinct roles. For example, LOTR-1, but not ZNFX-1, interacts with cytoskeletal proteins, *lotr-1* mutants are proficient in the inheritance of RNAi-induced silencing, and in *lotr-1* mutants the 5’ shift in 22G-RNA coverage is specific to WAGO/mutator targets, and hardly affects CSR-1 targets. Again, no additive effect on brood size was observed between the double and single mutants. These results add support to the hypothesis that physical interactions between LOTR-1 and ZNFX-1 are integral to one another’s primary function in Z granules–both acting to balance 22G-RNA distribution across WAGO/mutator targets (Fig 5D). The differences in phenotype cannot be currently explained, but we can speculate. One could think, for instance, about remaining ZNFX-1 function, even if ZNFX-1 does not localize in a germ granule in *lotr-1* mutants, whereas there would be no ZNFX-1 function at all in a *znfx-1* mutant. Whether this possibility is relevant, or whether there are other explanations, will have to be resolved with more experimentation.

We found that LOTR-1 colocalizes and coimmunoprecipitates with the Z-granule protein ZNFX-1 and that the two proteins interact in Y2H assays (Figs 1E, 1F, 3 and S3C). Associations between the two proteins are primarily maintained through LOTR-1’s Tudor domain (Figs 2A and S2A). Although global 22G-RNA levels remain the same in *lotr-1* mutants, these are deregulated and altered within specific subpopulations (Figs 4A, 4B, S5A and S5B). Like *znfx-1* mutants, 22G-RNA coverage of WAGO/mutator targets show a 5’ shift, particularly on up-regulated transcripts (Figs 4C–4E and S5C–S5F). However, unlike *znfx-1*, in *lotr-1* mutants this 5’ shift extends only to WAGO/mutator targets and not to CSR-1 targets (S5E–S5H Fig). A second difference between *lotr-1* and *znfx-1* mutants relates to the inheritance of RNAi across generations. In *znfx-1* mutants, RNAi inheritance is defective, whereas in *lotr-1* mutants it is enhanced. In fact, we observed inheritance for more generations in *lotr-1* mutants than in wild type animals. Clearly, ZNFX-1 and LOTR-1 are not simply co-factors that require each other’s presence. The differences in phenotype may be related to the possibility that in *lotr-1* mutants ZNFX-1 is still functional, whereas in *znfx-1* mutants all its activity is lost. A much better understanding of the molecular functions of these proteins, and the relevance of their presence in germ granules will be required to fully understand these differences in phenotype.

Another outstanding question is the functional relationship between LOTR-1, which combines its two LOTUS domains with a Tudor domain, and the recently discovered paralogs MIP-1 and MIP-2, which each contain two LOTUS domains and long intrinsically disordered regions. Y2H assays show that both MIP-1 and MIP-2 associate with GLH-1/Vasa in P granules [72], while Y2H does not support a direct interaction between LOTR-1 and GLH-1 (S3B Fig). The association of MIP-1 and MIP-2 with GLH-1 in *C*. *elegans* is likely similar to Oskar’s association with Vasa in *Drosophila*. [14,16] Interestingly, we show that the C-terminus of LOTR-1 also associates with the C-terminus of both MIP-1 and MIP-2 via Y2H (S3A Fig), and that MIP-2 is enriched in both ZNFX-1 and LOTR-1 IP-qMS from young adult lysates (Fig 3). Depletion of LOTR-1 or both of the MIPs can reciprocally impact each other’s association with germ granules (Figs 2G, 2H and S2J–S2L). This complicates labeling these proteins as strict components of either P- or Z- granules, implying a degree of inter-granule crosstalk. It also suggests there may be partial synergy among these three LOTUS-domain proteins. If *C*. *elegans* LOTUS domain proteins promote RNA helicase activity in a manner similar to the activation of *Drosophila* Vasa by Oskar [14,16,88], one possibility is that the presence of multiple LOTUS domain proteins in P granules provides some level of redundancy for the small RNA biogenesis machinery. For example, the increased association of MIP-2 with ZNFX-1 in *lotr-1* mutant young adults could partially compensate for the absence of LOTR-1. It remains to be determined whether the association of MIP-1 and MIP-2 with GLH helicases in P granules is analogous to the association of LOTR-1 with the ZNFX-1 helicase in Z granules, and whether these P- and Z-granule associations distinguish 22G-RNA distribution across CSR-1 and WAGO/mutator targets.

GLH-1, DEPS-1, PRG-1, PGL-1, and PGL-3 are all constitutive P-granule components, and thus far they have not been observed to colocalize with Z granules; however, both PRG-1 and DEPS-1 were significantly enriched by LOTR-1 and ZNFX-1 IP-qMS (Fig 3). While this may reflect associations in early embryo germline blastomeres, before P and Z granule demixing in PGCs [70], it also strengthens the likelihood of dynamic inter-granule crosstalk in PGCs and in germ cells during larval and adult development. Interestingly, WAGO-3 and SMG-2 associations with both ZNFX-1 and LOTR-1 are only significant in embryo lysates, despite their abundant mRNA expression in adults (Fig 3C and 3F). In contrast, the association between LOTR-1 and MEG-3 corresponds with MEG-3 expression in early embryos (Fig 3C). MEG-3 is an embryo-specific scaffold protein that nucleates germ-granule assembly after fertilization in the one-cell zygote. [89–92] The interaction with MEG-3 is dependent on the Tudor domain of LOTR-1 and implicates MEG-3 in the initial localization of LOTR-1 to germ granules. The LOTUS domains of LOTR-1 may keep the MEG-3 association in check, as the LOTR-1 association with MEG-3 increases when the LOTUS domain is deleted.

Additional insight into the contribution of LOTR-1’s LOTUS and Tudor domains can be inferred from domain-specific deletions. IP-qMS data show that the association of germ granule proteins PRG-1, WAGO-1, WAGO-4, DEPS-1 and ZNFX-1 with LOTR-1 *in vivo* is lost upon deletion of either its LOTUS or Tudor domains (Fig 3). The latter result is consistent with the observation that LOTR-1 is dispersed from germ granules in the absence of the Tudor domain (and thus less likely to encounter other germ granule components); however, LOTR-1 localization to germ granules is independent of its LOTUS domains (Figs 2A and S2A). This suggests that the association of LOTR-1 with these germ-granule proteins depends at least partially on specific interactions with its LOTUS domains. LOTR-1 associations that depend specifically on its LOTUS domain, based on IP-qMS data, include the actin-binding proteins HUM-1, HUM-5, CLIK-1, and GSNL-1, as well as 3’UTR associated proteins SUF-1 and CPF-1 (Fig 3). Therefore, the LOTUS domain may be used to tether LOTR-1 to the cytoskeleton and 3’UTR of Z granule-associated transcripts, providing potential mechanisms for Z-granule demixing or the ability to counter the 5’ coverage bias of WAGO 22G-RNA targets during the amplification of small RNAs. In fact, the most significant impact on Z granule composition in *lotr-1* mutants is decreased association of SUF-1 and CPF-1 (Fig 3F). This suggests that RdRP-dependent 22G-RNA amplification along WAGO/mutator targets may be enriched within the more 3’ regions of the targeted transcript by the interaction between LOTR-1 and the 3’ UTR.

In *Drosophila*, *tejas/tapas* double mutant males are infertile. [33,34] Similarly, in mice, loss of either TDRD5 or TDRD7 will cause male-specific sterility, with defects during spermiogenesis. [31,32,93,94] Even though LOTR-1 does not affect piRNA biogenesis in *C*. *elegans*, our study reveals an intriguing parallel to mouse mutants lacking TDRD5 (Fig 4). The loss of 22G-RNAs from the 3’ regions of 22G-RNA producing loci in *lotr-1* mutants resembles the loss of piRNAs from the 3’ regions of (pachytene) piRNA producing loci in Tdrd5 mutant mice (Figs 4A–4C and S5). [28] It will be interesting to test if mammalian ZNFX1 acts together with TDRD5, even to the extent of producing Z-granule-like molecular condensates, and to resolve the mechanism that maintains small RNA production over the complete length of small RNA producing loci. Given our identification of 3’ processing factors in association with LOTR-1 and ZNFX-1, an intriguing hypothesis would be that LOTR-1 and ZNFX-1 target the small RNA producing machinery, be it RdRP in *C*. *elegans*, or specific nucleases in mammals, to the 3’ ends of transcripts through interactions with 3’ end processing complexes (Fig 5D).

## Materials and methods

### Sequence, structural, and evolutionary analysis

To characterize LOTR-1 and its interactions, we used sequence analysis (PSI/Delta-BLAST, HHPred, and multiple sequence alignment with PRALINE) to identify conserved protein domains. [95–98] Homology modeling was performed with RaptorX: eLOTUS domain was constructed from Oskar LOTUS (PDB ID: 5NT7); mLOTUS domain from Cdt1 (PDB ID: 5MEC), which contains winged-helix domains that interact with subunits of the MCM helicase motor; and Tudor domain from TD3 (PDB ID: 4b9wA) from TDRD1.

Conservation of LOTR-1 in nematodes was analyzed as previously described. [99] Proteomes of nematode species were downloaded from WormBase ParaSite (WBPS16) and BLAST databases were created from each proteome with makeblastdb–dbtype prot. Then, two sets of whole-proteome BLASTP searches were performed: 1) using the *C*. *elegans* proteome as query versus all other proteomes; and 2) the reciprocal BLASTPs using all the proteomes as query versus the *C*. *elegans* proteome. From the first set of BLASTP searches, we extracted the best BLAST hit for *C*. *elegans* D1081.7/LOTR-1 (full length) in other species. We used the best BLAST hits, to get a bit score from the reciprocal BLASTP (second set of BLASTPs) when D1081.7/LOTR-1 was the best reciprocal BLASTP hit. The bit scores were normalized by dividing by the *C*. *elegans* bit score. When no reciprocal BLAST hit was found, the score of 0 was given. The scores were loaded into R v.4.1.0 and plotted using publicly available packages (ggplot v.3.3.5, using geom_tile()). Nematode clades were annotated similar to Rodrigues et al., 2019. [99]

### Strain generation and maintenance

*C*. *elegans* strains were maintained using standard protocols. [100] A complete list of strains and alleles generated for this study is provided (S4 Table). CRISPR/Cas9 was used to place tags on endogenous genes and generate mutant alleles as described. [101] All alleles generated for this study were sequence verified.

### Imaging

Live worms were mounted on agarose pads in egg buffer (25 mM HEPES, 120 mM NaCl, 2 mM MgCl2, 2 mM CaCl2, 50 mM KCl, and 2 mM levamisole), covered by No. 1.5 coverslips, and imaged with a Leica DMI6000B widefield epifluorescence microscope equipped with a Leica HC PL APO 63x/1.47NA oil immersion objective. Samples were excited with a Leica EL6000 light source and EGFP (11525314) and Texas Red filter (11525310) sets. Images were acquired with a Leica DFC9000GT camera controlled with LAS acquisition software with the same conditions, 2048X2048 pixel and in 16 bits. Figs 1D, 2A–2F and S2F and S2I are deconvolved 30μm projections of the germline loop region. For quantification in S2A,B,D,E,G and H, a single-plane was acquired. Fig 1E images are 8μm and 1μm maximum intensity projections of pachytene germ cells acquired with an upright Zeiss LSM 980 confocal microscope (axio examiner) equipped with AiryScan2 detector and a Zeiss Plan Apochromat 63X/1.4NA oil immersion objective, mounted on a piezo insert and controlled by Zen Blue 3.1 acquisition software. RFP and EGFP fluorescence were respectively excited with the 561nm (2.5% laser power) and 488 nm (1.50% laser power) laser line and the emission were collected from 573 to 627nm and from 499 to 557nm. A 8um Z-stack series optical sections were collected sequentially, in bidirectional mode, with a pixel size of 0.043um X 0.043um, a step-size of 0.17 um, at zoom 12, in 16 bit and in Super Resolution mode (SR). Images were processed with a strength of the deconvolution set to 8.5 or 6.7 for EGFP and 8.1 and 6.3 for RFP 8.1 and saved in CZI file[102]. Image analysis was performed using the open-source FIJI software. [102]

Center of mass differences were calculated from 20x40 micron red/green sections from single-plane images of pachytene cells, 1 image/worm, 20 worms/strain. A FIJI macro (included in S5 Table) was applied that uses a Difference of Gaussian (DoG) algorithm to identify and mask germ granules in each channel (https://micro.magnet.fsu.edu/primer/java/digitalimaging/processing/diffgaussians/index.html). The JACoP plugin was used to identify the geometrical center for each germ granule and then calculate the ‘distance-based colocalization’ with its nearest neighbor in the corresponding channel. [103] S5 Table contains the distances between granule pairs in each of the twenty images from both strains that were examined.

Germ-granule size and number were calculated from 20x40 micron sections from single-plane images of pachytene, oocyte, or spermatogenesis regions, 1 image/worm, 20 worms/strain. To do this, an ImageJ ‘germ-granule counter’ macro (include in S5 Table) was applied to ‘MaxEntropy AutoThreshold’ granules and ‘Analyze Particles’, providing granule number and size in each 20x40 section. S5 Table contains granule number and size data for the graphs in S2 Fig. RFP::ZNFX-1, DEPS-1::GFP, MIP-1::GFP, and MIP-2::GFP levels in S3H Fig were calculated from the average pixel intensity of 20x40 micron sections from single-plane images of the pachytene region, 1 image/worm, 20 worms/strain.

### RNAi

RNAi feeding was performed as previously described. [104] The L4440 plasmid in HT115 bacteria was used as the RNAi control. RNAi was performed starting on L1 worms, with at least three biological replicates, and used feeding constructs previously described. [105] For simultaneous depletion of *mip-1* and *mip-2*, equal parts from the Ahringer RNAi feeding library clones were mixed and fed to L1 worms.

*pos-1 RNAi resistance assays*: 10 L3 stage animals were singled onto *pos-1* RNAi feeding plates. After two days, adults were removed and eggs were counted, and the number of hatched larvae counted the following day. Data were analyzed with a generalized linear mixed effects model for Bernoulli-distributed binary data using the R package *lme4*. [106] The log-odds-ratio of hatched larvae was modeled as a linear combination of coefficients for three factors: the focal factors *genotype* and *RNAi treatment* were modeled as fixed effects, while *plate membership* was included as a random effect to account for the (in some cases) considerable differences in hatching frequencies between plates of the same genotype-condition group. Likelihood ratio tests were used to determine significance of differences in RNAi treatment effect between WT and *lotr-1(xf60)* and between WT and *lotr-1(sam117)*. 95% confidence intervals for the hatching probabilities of the respective genotype-treatment groups (excluding random plate effects) were calculated by parametric bootstrapping (bootstrap size 10000).

*Transgenerational epigenetic inheritance (TEI) assays*: Two sets of experiments were performed in different laboratories to test TEI.

For Fig 5B, Worms expressing GFP::H2B (*mjIS31*) in wild-type N2 and *lotr-1(xf60)* mutants were bleached and embryos were placed on *E*. *coli HT1115 gfp* RNAi or control L4440 plates. [50,86] After four days, adult RNAi treated P0 worms were screened for presence or absence of GFP expression. The RNAi treated P0 worms were then bleached and F1 embryos were placed onto OP50 plates. Four days later F1 worms were screened for GFP expression. We considered worms that expressed GFP at levels comparable to non-RNAi treated worms as GFP expressing, and worms that were either completely silenced or had barely detectable expression of GFP as silenced. To score GFP expression we used a Leica Thunder Imager Live Cell microscope with a HC FLUOTAR L VISIR 25x/0.95 water objective. Representative images were taken with a Leica AF7000 microscope with a sCMOS Flash 4.0 Hamamatsu camera and a HCX PL APO 40x/1.1 water objective using LASX Application Suite X 3.7.4.23463 software. Image J v. 1.53c was used for visualization. For microscopy sample preparation, approximately 50 worms were washed in a drop of M9 buffer and subsequently transferred to a 50 μl drop of 40mM sodium azide in M9 buffer on a coverslip. Once the worms were paralyzed, excess buffer was removed and a glass slide with a 2% (w/v) Agarose pad was placed on top of the coverslip.

For Fig 5C, L1 animals expressing H2B::GFP (*ruIs32*) in wild-type N2 and *lotr-1(usa1)* mutants were placed on GFP(RNAi) and control L4440(RNAi) plates at 20°C. 100 progeny from each plate were scored for GFP expression in the germ line using a Leica DM5500 widefield microscope with a 40X objective, and the Leica LASX software. From the GFP(RNAi) plates, ten silenced young adults from both wild type and *lotr-1* mutants were singled to individual plates seeded with OP50 on NGM plates (referred to as “F1 generation” in Fig 5C). For each line, the progeny (n>50) was scored for GFP expression. Lines were recorded as still silenced if no GFP or only weak GFP expression was detected. Silenced lines were propagated by moving 3 young adults into a fresh NGM plate every new generation, and scored for GFP expression for a maximum of ten successive generations. Data from three replicate experiments were pooled and the proportion of silenced lines in each generation, out of a total of 30 individual lines, was plotted. Kaplan-Meier curves were fitted to assess survival and significance tested with a log-rank test.

### Brood size

Brood sizes were counted from each strain for S2C Fig after placing ten or more L4 worms on individual plates at 20°C and 26°C. For the generational brood size assay in S4B Fig, 5 L4 worms from each strain were cloned to individual plates and shifted from 20°C to 25°C. Worms were kept at 25°C for 5 generations, with L4 progeny from each plate used to start the subsequent generation.

### Germline mortality

Worms were assessed for the mortal germline phenotype using the assay described in Ahmed et al. 2000. [107] Three *lotr-1* strains (with the *xf58*, *xf60*, *xf61* alleles) and wild-type N2 animals were synchronized by bleaching and overnight hatching in M9 and L1-arrested animals. Five L1-L2 worms were picked to a new plate and grown at 25°C. The sixth day L1-L2 worms, corresponding to the third generation from the picked worms, were again hand-picked to a new plate. Fertile generations were counted until sterility ensued and no progeny could be isolated. Five replicates were used in this assay, N2 worms were used as control. Worms were always picked before starvation to avoid any effect it might cause during the assay, including extension of transgenerational germline lifespan. [108]

### Yeast two hybrid

Full-length or truncated cDNAs of *mip-1*, *mip-2*, *glh-1*, *lotr-1* and *znfx-1* and DNA encoding 3xFLAG tag were cloned in-frame with *GAL4-AD* or *GAL4-BD* into the multiple cloning site of pGAD-C1 and pGBD-C1. [109] Yeast cells from strain PJ69-4a were transformed with 1 μg of plasmid and carrier DNA with lithium acetate [110].Transformed cells were then plated on the appropriate drop-out media, and the presence of the newly introduced plasmids was checked by colony PCR. Bait-prey interactions were done on SC-Leu-Trp-Ade plates or SC-Leu-Trp-His plates supplemented with varying concentrations of 3-amino-1,2,4-triazole, as previously described [72].

### Reporters for piRNA activity and NRDE-3 localization

Experiments were performed as previously described. [45,63,86] Crosses (S4C-D) were set up in 6 cm Petri dishes with only 10 μl of OP50 to favor nematode meeting and mating. Wide-field fluorescence microscopy images (S4C-D, G) were acquired and processed with the Leica Application Suite (LAS) software (v3.1.0) on a Leica DM6000B.

### Tc1 reversion

Tc1 reversion experiments were performed as previously described. [111] Crosses were set up, and cross-progeny genotyped as described for the sensor experiments above. Twitcher *unc-22(st136)* worms homozygous for *lotr-1* were grown at 25°C: 24 independent populations of *lotr-1; unc-22(st136)* per *lotr-1* allele. Controls and *lotr-1* populations were grown in parallel for several generations, namely four plate passages, corresponding to 8–12 generations. Controls were grown in 2 independent populations for the same duration of time. Plates were screened every 2–3 days for revertants, i.e., animals that lost the twitching phenotype.

### Small RNA sequencing and bioinformatic analysis

#### Sequencing *wild-type* vs. *lotr-1(sam117)*

We collected *C*. *elegans* samples of wild-type and *lotr-1(sam117)*. Worms were synchronized by bleaching and overnight hatching in M9. L1 arrested worms were brought on OP50-seeded NGM plates. After 65 hours, gravid adult animals were washed off plates into fresh tubes with M9 buffer. Worms were washed with M9 buffer three times followed by three washes with Ultrapure water. Liquid was removed and 50 μl of worms were transferred into a new tube. For RNA extraction, 500 μl of Trizol LS (Life Technologies) was added to 50 μl of worms and frozen for 30 s in liquid nitrogen followed by thawing for 2 min at 37°C. Freeze-thaw cycles were repeated until worms were dissolved. Samples were centrifuged for 5 min at 14800g and supernatants were mixed 1:1 with cold 100% ethanol. RNA isolation was performed with Zymo Research Direct-zol RNA microprep kit (#R2062) according to manufacturer’s protocol and 1 μg RNA of each sample was subsequently treated with RNA 5´ Pyrophosphohydrolase (RppH, NEB) as described in [112]. NGS library prep was performed using the NEXTflex Small RNA-Seq Kit V3 following Step A to Step G of Bioo Scientific`s standard protocol (V19.01) with a ligation of the 3’ 4N Adenylated Adapter overnight at 20°C. Libraries were prepared with a starting amount of 580 ng RppH-treated RNA per sample, amplified in 16 PCR cycles, purified on an 8% TBE gel and size-selected in the range 144–163 bp. Purified libraries were profiled with a High Sensitivity DNA kit on a 2100 Bioanalyzer (Agilent), quantified using the Qubit dsDNA HS Assay Kit in a Qubit 2.0 Fluorometer (Life Technologies), pooled in equimolar ratio and sequenced on a NextSeq 500/550 Illumina sequencer.

#### Sequencing *wild-type* vs. *lotr-1(xf58/xf60/xf61)*

We collected *C*. *elegans* samples of wild-type, in triplicate, and 1x each *lotr-1* allele (1x *xf58*, 1x *xf60*, and 1x *xf61*). We subsequently treated these three *lotr-1* allele samples as triplicates. Worms were grown and collected similar to the method above, but gravid adults were collected 63 hours after plating. For RNA extraction, worms were thawed and M9 was replaced with 250 μL of worm lysis buffer (0.2M NaCl, 0.1M Tris pH 8.5, 50 mM EDTA, 0.5% SDS), supplemented with 300 μg Proteinase K (Sigma-Aldrich, P2308). Subsequently, lysis was conducted for approximately 90 minutes at 65°C. Next, 3 volumes of TRIzol LS (Life Technologies, 10296–028) were added and subsequent isolation was as defined by the manufacturer’s instructions. Samples were enriched for small RNAs using a mirVana Kit (Life Technologies, AM1561).

Samples were subsequently treated with RppH as described. [112] Then, 1 μg of RppH-treated RNA was loaded on a 15% TBE-urea gel and size-selected between 16- to 30-nt. Purified fraction was confirmed by Bioanalyzer sRNA chip (Agilent). Library preparation was based on the NEBNext Multiplex sRNA Library Prep Set for Illumina (New England BioLabs) with slight modifications. To counteract ligation biases, the 3’ and 5’ adapters contained four random bases at the 5’ and 3’-end, respectively, and were chemically synthesized by BioScientific. Adapter-ligated RNA was reverse-transcribed and PCR-amplified for 14 cycles using index primers. The PCR-amplified cDNA construct was purified using AMPure XP beads (Beckman Coulter). The purified PCR reaction was checked on the Bioanalyzer using High Sensitivity DNA chip (Agilent). Size selection of the sRNA library was done on LabChip XT instrument (Perkin Elmer) using DNA 300 assay kit. Only the fraction containing 140–165 bp was pooled in equal molar ratio. The resulting 10 nM pool was denatured to 10 pmol with 5% PhiX spike-in and sequenced as single-read on HiSeq 2500 Illumina sequencer.

#### Bioinformatic analysis

The raw sequence reads in FastQ format were cleaned from adapter sequences and size-selected for 18–30 base-long inserts (plus 8 random adapter bases) using cutadapt v.2.4 (http://cutadapt.readthedocs.org), with parameters ‘-a TGGAATTCTCGGGTGCCAAGG -m 26 -M 38’ (NextFlex samples) or ‘-a AGATCGGAAGAGCACACGTCT -m 26 -M 38’ (NEBNext samples), followed by quality checks with FastQC (http://www.bioinformatics.babraham.ac.uk/projects/fastqc), and MultiQC (https://multiqc.info/). Read alignment to the *C*. *elegans* genome (Ensembl WBcel235/ce11 assembly) with concomitant trimming of the 8 random bases was performed using Bowtie v.1.2.2 (http://bowtie-bio.sourceforge.net), with parameters ‘-v 1 -M 1 -y --best --strata --trim5 4 --trim3 4 -S’ and the SAM alignment files were converted into sorted BAM files using Samtools v.1.9 (http://www.htslib.org). WBcel235/ce11 gene annotation in GTF format was downloaded from Ensembl release 96 (ftp://ftp.ensembl.org/pub/), and annotation for transposable elements (LINE, SINE, LTR, DNA and RC) in GTF format was downloaded from the UCSC Table Browser (http://genome-euro.ucsc.edu/cgi-bin/hgTables) RepeatMasker track and merged with the gene annotation GTF file using the Ensembl chromosome naming style. Aligned reads were assigned to individual small RNA loci and classes using Samtools, GNU Awk, Bedtools v.2.27.1 (http://bedtools.readthedocs.io), and Subread featureCounts v.1.6.2 (http://subread.sourceforge.net/), based on the following criteria: structural reads map sense to rRNA, tRNA, snRNA and snoRNA loci; miRNA reads are between 21 and 24 bases and map sense to mature miRNA loci; 21U-RNA reads are 21 base long, start with T and map sense to piRNA (21ur) loci; 22G-/26G-RNA reads are 22/26 base long, start with G and map antisense to transposons or to exons of protein-coding genes, lincRNAs and pseudogenes. Locus-level differential expression analyses of the small RNA classes (miRNA, 21U-RNA, 22G-RNA and 26G-RNA) were carried out using the Bioconductor package DESeq2 v.1.30.0 (https://bioconductor.org/packages/release/bioc/html/DESeq2.html), using a significance cutoff of 10% FDR and 2-fold change. Normalized 22G-RNA coverage tracks in bigWig format were produced using Bedtools and kentUtils v.385 (https://github.com/ucscGenomeBrowser/kent), using custom normalization based on the size factors computed by DESeq2. Relative metagene plots of 22G-RNA read coverage over selected gene sets were prepared using a previously described custom normalization strategy [71]. Selected gene sets were extracted from the GTF gene annotation from Ensembl. Then, 22G-RNA coverage for each gene-set was computed using deepTools v.3.1.0 (https://deeptools.readthedocs.io) with parameters ‘computeMatrix scale-regions --metagene --transcriptID gene --transcript_id_designator gene_id --missingDataAsZero --outFileNameMatrix’. Finally, using R v.4.0.3 the resulting count matrix of every sample was filtered keeping genes with rowSums above 10, 22G-RNA counts for each row(gene) were individually normalized using ‘rowSums’ and cumulative profiles computed by ‘colSums’ were plotted as representative metagene plots of relative 22G-RNA coverage over gene transcripts divided into 100 bins from 5’-TSS to 3’-TES. Metagene plots of total 22G-RNA coverage were computed the same way but without rowSums filtering and individual normalization per gene. Analyzed by metagene plots were genes with up-regulated (>2-fold at 10% FDR), down-regulated (>2-fold at 10% FDR) or unaffected (p = 1 and baseMean>10) 22G-RNA levels, as well as genes reported as CSR-1 [56], WAGO [46], mutator [69], ALG-3/4 [63] or ERGO-1 [63] targets. WormMine database (http://intermine.wormbase.org/tools/wormmine/begin.do) was utilized for gene identifier conversion. Genome tracks of normalized 22G-RNA coverage at selected genes were plotted using R v.4.1.0 with the Bioconductor packages Gviz v.1.36.2 (https://bioconductor.org/packages/release/bioc/html/Gviz.html) and GenomicFeatures v.1.44.2 (https://bioconductor.org/packages/release/bioc/html/GenomicFeatures.html). Sequencing data have been deposited to the NCBI Gene Expression Omnibus (GEO) under accession number GSE192794.

### α-FLAG Immunoprecipitation and Western Blot

Immunoprecipitation and mass spectrometry were performed largely as described. [63] Animals were grown either in normal OP50-seeded NGM plates or on OP50 high-density plates. The protocol for the production of the latter was adapted from (Schweinsberg and Grant, 2013, https://www.ncbi.nlm.nih.gov/books/NBK179228/). In short, commercially available chicken eggs were cracked, the yolks isolated and thoroughly mixed with LB medium (50 mL per egg yolk). Then, the mix is incubated at 65°C for 2–3 hours. After cooling down, pre-grown OP50 liquid culture is added to the mix (10 mL per egg). 10 mL of this preparation is poured into each 9 cm NGM plate and plates are decanted the next day. After 2–3 days of further bacterial growth and drying, plates should be stored at 4°C.

Worms expressing 3xFLAG-tagged LOTR-1 and ZNFX-1 were grown and synchronized by bleaching and overnight hatching in M9. Synchronized young adults with no visible embryos were collected 51–56 hours post-plating, washed in M9, followed by a last wash in water, and frozen in dry ice. Prior to IP, samples were thawed, 2x Lysis buffer was added (50 mM Tris/HCl pH 7.5, 300 mM NaCl, 3 mM MgCl_2_, 2 mM DTT, 0.2% Triton X-100, 1 complete Mini protease inhibitor tablet, #11836153001) and lysis was conducted with sonication in a Bioruptor Plus (Diagenode, on high level, 10 cycles of 30 seconds on/off). Embryos were collected by bleaching gravid animals approximately 72 hours after plating, washing with M9, plus one last washing step in 1x lysis buffer (25 mM Tris/HCl pH 7.5, 150 mM NaCl, 1.5 mM MgCl_2_, 1 mM DTT, 0.1% Triton X-100, 1 complete Mini protease inhibitor tablet, #11836153001) and by freezing worm pellets in liquid N_2_ in a pre-cooled mortar. Prior to IP, the pellets were ground to a fine powder in a pre-cooled mortar, then transferred to a cold glass douncer and sheared for 40 strokes with piston B. First round of IPs to FLAG-tagged LOTR-1 were performed using 1.5 mg of total embryo or young adult extracts, while for the second round of these experiments 1 mg of embryo or young adult extracts were used. IPs to FLAG-tagged ZNFX-1 were performed using 1 mg of young adult extract and 0.65 mg of embryo extract. IPs were performed in quadruplicates, with exception of FLAG IPs to LOTR-1(ΔTudor) in embryos, which were conducted in triplicate. FLAG-tag immunoprecipitation was performed using an α-FLAG antibody (Monoclonal ANTI-FLAG M2 antibody produced in mouse, Sigma Aldrich, #F3165) and Protein G magnetic beads (Invitrogen Dynabeads Protein G; #10004D). 30 μL of beads were used per IP. Beads were washed three times with 1 mL of Wash Buffer (25mM Tris/HCl pH 7.5, 300 mM NaCl, 1.5 mM MgCl_2_, 1 mM DTT, 1 complete Mini protease inhibitor tablet, #11836153001). 2 μg of α-FLAG antibody were incubated with the beads and the extract for approximately 3 hours, rotating at 4°C. Afterwards, samples were washed 5 times with 1 mL of Wash Buffer. Finally, the beads were resuspended in 1x LDS buffer (Thermo Scientific, #NP0007) supplemented with 100 mM DTT and boiled at 95°C for 10 minutes.

Samples for mass spectrometry were prepared in quadruplicates: four lysates were prepared from pelleted young adult worms or pelleted embryos for each strain. Independent anti-FLAG IP-qMS experiments were then performed on each of these lysates. This same process was then duplicated on a separate occasion with new young adult and embryo lysates, again using quadruplicates.

Western blot in S3D Fig was performed exactly as described [113] using a mouse monoclonal anti-FLAG antibody (Sigma-Aldrich, #F3165) in a 1:5000 dilution in Skim Milk solution (1x PBS, 0.1% Tween-20, 5% (w/v) Skim Milk Powder), and a rabbit anti-Actin antibody (Sigma-Aldrich, #A2066) in a 1:500 dilution in Skim Milk Solution. We first probed the IP bait with a HRP-linked secondary antibody (Cell Signaling Technology, anti-mouse IgG, #7076; 1:10,000 dilution in Skim Milk Solution), and Actin was subsequently probed with a LI-COR secondary antibody (LI-COR IRDye 800CW Donkey anti-Rabbit IgG (H+L), #926–32213, in a 1:15,000 dilution in Skim Milk Solution). 1.5 mg of young adult extract were used for LOTR-1 anti-FLAG IPs, while 1 mg of young adult extract were used for ZNFX-1 anti-FLAG IPs.

The western blot in S3E Fig was performed as above using fed and synchronized young adults washed from two plates (approximately 1000 worms/sample), flash frozen in 50 μl of 2X Laemmli sample buffer (65.8mM Tris-HCl, pH 6.8, 2.1% SDS, 26.3% (w/v) glycerol, 0.01 bromophenol blue). Samples were run on a 4–15% gradient gel (BioRad), simultaneously probed with anti-FLAG antibody (1,5000) and a mouse anti-tubulin antibody (Sigma-Aldrich, #T9026 in a 1:3000 dilution). FLAG and tubulin were detected with HRP-linked secondary (goat anti-mouse antibody Invitrogen #31430 in a 1:3000 dilution). ImageJ was used to measure relative expression levels of LOTR-1 in relation to tubulin.

The Western blot in S3F Fig was performed using extracts derived from roughly 8000 synchronized young adult animals raised on the appropriate RNAi plates. Collected animals were cleared of the bacteria and flash frozen in RIPA buffer (150 mM NaCl, 1% v/v Triton X-100, 0.5% w/v sodium deoxycholate, 0.1% w/v sodium dodecyl sulfate, and 0.05 M Tris-HCl pH 7.5) supplemented with 1% v/v protease inhibitors (Sigma P8340), then homogenized with 425–600 μm diameter glass beads (Sigma G8772). Westerns were run with 80 μg of supernatants per lane on 4–12% bis-tris gels (Thermo Fisher Scientific NP0335) and simultaneously probed with anti-FLAG (Sigma clone M2 F3165, 1:4000) and anti-tubulin (DSHB clone 12G10, 1:10000), then detected with an HRP-linked secondary serum (GE NXA931, 1:10000). ImageJ was used to measure relative expression levels of LOTR-1 in relation to tubulin.

### Mass spectrometry

IP samples were boiled at 70°C for 10 minutes and separated on a 4–12% gradient Bis-Tris gel (Thermo Fisher Scientific, #NP0321) in 1x MOPS (Thermo Fisher Scientific, #NP0001) at 180 V for 10 minutes. Then, samples were processed separately, first by in-gel digestion [114,115], followed by desalting with a C18 StageTip. [116] Afterwards, the digested peptides were separated on a heated 50-cm reverse-phase capillary (75 μm inner diameter) packed with Reprosil C18 material (Dr. Maisch GmbH). Peptides were eluted along a 90 min gradient from 6 to 40% Buffer B (see StageTip purification) with the EASY-nLC 1,200 system (Thermo Fisher Scientific). Measurement was done on an Orbitrap Exploris 480 mass spectrometer (Thermo Fisher Scientific) operated with a Top15 data-dependent MS/MS acquisition method per full scan. All raw files were processed with MaxQuant (version 1.5.2.8) and peptides were matched to the *C*. *elegans* Wormbase protein database (version WS269). Differential enrichment was defined for 3xFLAG::GFP::LOTR-1 IPs versus 3xFLAG::GFP::*lotr-1(deletion)* IPs. Venn diagrams were produced with Venny 2.1 (https://bioinfogp.cnb.csic.es/tools/venny/). Raw data and detailed MaxQuant settings can be retrieved from the parameter files uploaded to the ProteomeXchange Consortium via the PRIDE repository: identifier PXD025186.

## Supporting information

S1 Fig Sequence conservation of LOTUS and Tudor domains across species. A) Conservation of LOTR-1 in nematodes showing a normalized score for the best reciprocal BLASTP hit. B-C) Sequence alignment across indicated species for the B) LOTUS domains and C) Tudor domains. D) Conservation of LOTUS and Tudor domains across indicated proteins and species.
(TIF)

S2 Fig Germ-granule phenotypes and quantification. A) Comparison of LOTR-1 granule number and size in the pachytene region of *lotr-1* mutants. B) Comparison of LOTR-1 and PRG-1 granule number and size during spermatogenesis in *lotr-1* mutants. C) Brood size at permissive (20°C) and restrictive (26°C) temperatures in indicated *lotr-1* mutants. PRG-1 granule number and size in D) pachytene and E) oocyte regions of *lotr-1* and/ or *znfx-1* mutants. F) mCherry::PRG-1 and GFP::LOTR-1 distribution during spermatogenesis in both the presence and absence of LOTR-1’s Tudor domain. ZNFX-1 granule number and size in G) pachytene and H) oocyte regions of *lotr-1* mutants. I) Comparison of GFP::LOTR-1 and mCherry::PRG-1 expression in the germlines of first generation *znfx*-1 mutant worms. J) Normalized granule counts in the pachytene region of GFP-tagged LOTR-1, MIP-1, and MIP-2 granules following empty vector control and *mip-1;mip-2* RNAi (top), and in wild type and a *lotr-1* deletion allele (bottom). Normalized granule counts in the K) pachytene and L) oocyte regions of GFP-tagged LOTR-1, GLH-1, PGL-3, DEPS-1, MIP-1, and MIP-2 granules following empty vector control and *lotr-1* RNAi. P-values from unpaired t-tests, where p>0.05 is ns, p<0.05 is *, p<0.01 is **, p<0.001 is ***, and p<0.0001 is ****.
(TIF)

S3 Fig LOTR-1 associates with the MIP proteins and ZNFX-1, but not GLH-1 by Y2H, and anti-FLAG IPs. A) Y2H analysis of MIP-1, MIP-2, and LOTR-1. MIP-1’s C-terminal half interacts with full length LOTR-1. LOTR-1’s C-terminal half interacts with both MIP-1 and MIP-2, and the LOTR-1/MIP interactions are independent of LOTR-1’s LOTUS domains. B) Y2H analysis of LOTR-1, MIP-1 and GLH-1 do not uncover an interaction between LOTR-1 and GLH-1. C) Y2H activation through the N-terminal third of ZNFX-1 is strengthened by an association with LOTR-1. D) Western blot for LOTR-1 immunoprecipitations with strains and anti-FLAG antibody used for IP-qMS. E) Western blot showing 3xFLAG::GFP::LOTR-1 expression in mutant backgrounds, α-tubulin loading control. F) Western blot showing 3xFLAG::GFP::LOTR-1 expression following RNAi, α-tubulin loading control. Mean RFP::ZNFX-1 expression intensity in the pachytene region of *wild-type* and *lotr-1* mutants. G) ZNFX-1 immunoprecipitations with the strains and anti-FLAG antibody used for IP-qMS. Done using synchronized young adult worms. Mutations in *lotr-1* are indicated above the blots. H) Mean fluorescence intensity of tagged ZNFX-1, DEPS-1, MIP-1 and MIP-2 in the pachytene region of control RNAi, *lotr-1* RNAi, and *mip-1; mip-2* RNAi. P-values from unpaired t-tests, where p<0.001 is ***, and p<0.0001 is ****.
(TIF)

S4 Fig The effect of *lotr-1* mutations of small RNA silencing and transposon mobilization. A) Number of fertile lines of each strain indicated per generation. The onset of sterility of *lotr-1* mutants occurred at the 18^th^ generation in the *xf58* and *xf60* alleles, and at the 20^th^ generation in the *xf61* allele. The decline in fertility proceeded in all *lotr-1* strains until no fertile line remained. B) Brood sizes at 25°C in *lotr-1* and *znfx-1* mutants over five generations. A T-test was used to determine assess significance at generation five. C) Cross scheme of a non-stably silenced 21U-RNA sensor with *lotr-1(xf58)* mutants. The F3 of the indicated genotype was scored for mCherry expression in the germline, bottom shows photomicrograph example. D) Schematics of two independent crosses between two *lotr-1* mutant alleles and a 21U-RNA sensor that is stably silenced under RNAe, bottom shows representative photomicrograph. E) Schematic of the *unc-22(st136)* allele and of the crosses performed. In an otherwise wild-type background, the Tc1 copy integrated in the *unc-22* gene does not mobilize, and these mutants display a twitcher phenotype. However, if transposon silencing is compromised Tc1 will become mobile and transpose leaving an intact *unc-22* gene, which restores the wild-type phenotype. Layout of the *unc-22(st136)* x *lotr-1* crosses to address Tc1 derepression is also shown. No events of phenotypic reversion were found in *lotr-1* mutants. F) Differential interference contrast (DIC), and fluorescence photomicrographs of embryos and L4 animals of the indicated genotypes. GFP::NRDE-3 is observed in the nuclei of hypodermic seam cells, indicated by white arrowheads. The images are representative of at least 10 embryos or 10 L4 worms. Scale is 20 microns. G) Genome tracks showing normalized 22G-RNA levels in the *eri-6/7* locus of wild type and *lotr-1(sam117)* animals.
(TIF)

S5 Fig 22G-RNA deregulation and distribution over gene classes in *lotr-1* mutants. A-B) Bar plots depicting the fraction of genes with up- or down-regulated 22G-RNAs in *lotr-1(sam117)* mutant belonging to the indicated gene classes (A) and target gene lists (B) defined in previous studies. C-D) Genome tracks of 22G-RNA coverage in wild type (N2) and *lotr-1(sam117)* mutant depicting two representative genes showing down-regulation (C) and up-regulation (D), as well as 5’ shift in 22G-RNA distribution. E-F,H-J) Metagene plots to visualize the relative 22G-RNA distribution in wild-type (N2) and *lotr-1(sam117)* mutant over target gene sets of WAGO (E), mutator (F), CSR-1 (H), ALG-3/4 (I) or ERGO-1 (J). TSS/TES, transcription start/end site. G) Genome tracks of 22G-RNA coverage of *Turmoil2*, a transposable element located within an intron of the gene *fbxa-192* targeted by 22G-RNAs, in wild-type (N2), upper track, and *lotr-1(sam117)* mutant, lower track. K) Differential analysis MA plot of 22G-RNA changes in three different *lotr-1* alleles (*xf58*, *xf60*, *xf61*) with partial deletions in the *lotr-1* gene. The vast majority (77%) of the deregulated genes highlighted in red color (>2-fold at 10% FDR) are also similarly affected in the *lotr-1* (*sam117*) null mutant shown in Fig 4B. L-M) Relative metagene plots over WAGO and mutator target genes in wild-type (N2) and *lotr-1*(*xf58/xf60/xf61*) mutants show very similar profiles to *lotr-1*(*sam117*) mutant.
(TIF)

S1 Table Enriched Proteins of 3xFLAG::GFP::LOTR-1 anti-FLAG IP-qMS. (XLSX)

S2 Table Enriched Proteins of 3xFLAG::GFP::ZNFX-1 anti-FLAG IP-qMS. (XLSX)

S3 Table 22G-RNA and 26G-RNA differential expression analysis with gene annotation. (XLSX)

S4 Table Strains used or created for this study. (XLSX)

S5 Table Supporting data. (XLSX)
