# Supplementary figures and images for: The Caenorhabditis elegans TDRD5/7-like protein, LOTR-1, interacts with the helicase ZNFX-1 to balance epigenetic signals in the germline
Source: PLoS Genet. 2022 Jun 3;18(6):e1010245. doi: 10.1371/journal.pgen.1010245 (PMC9200344; doi:10.1371/journal.pgen.1010245)

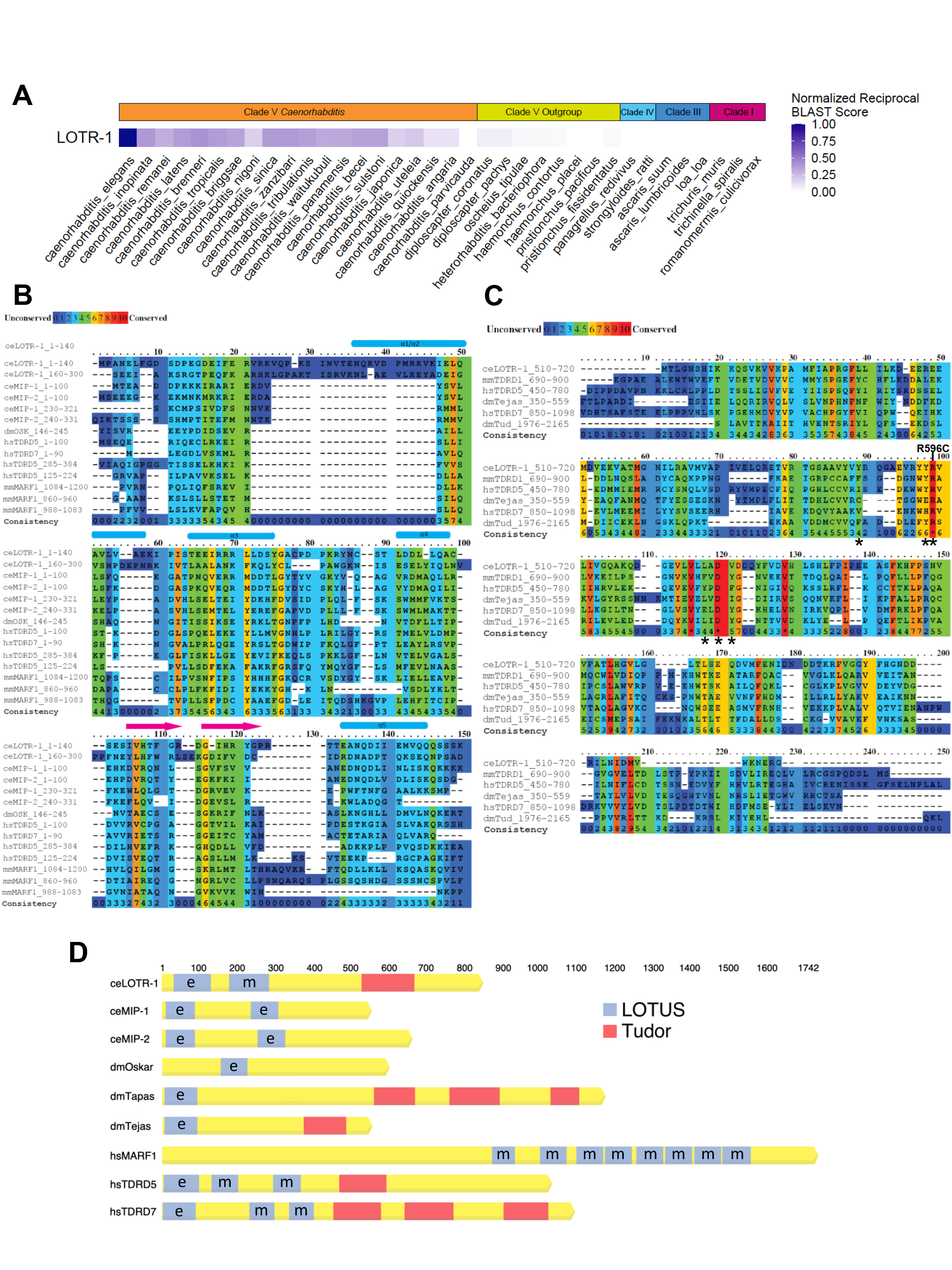

Supplement: S1 Fig — A) Conservation of LOTR-1 in nematodes showing a normalized score for the best reciprocal BLASTP hit. B-C) Sequence alignment across indicated species for the B) LOTUS domains and C) Tudor domains. D) Conservation of LOTUS and Tudor domains across indicated proteins and species. (TIF) [file pgen.1010245.s001.tif]

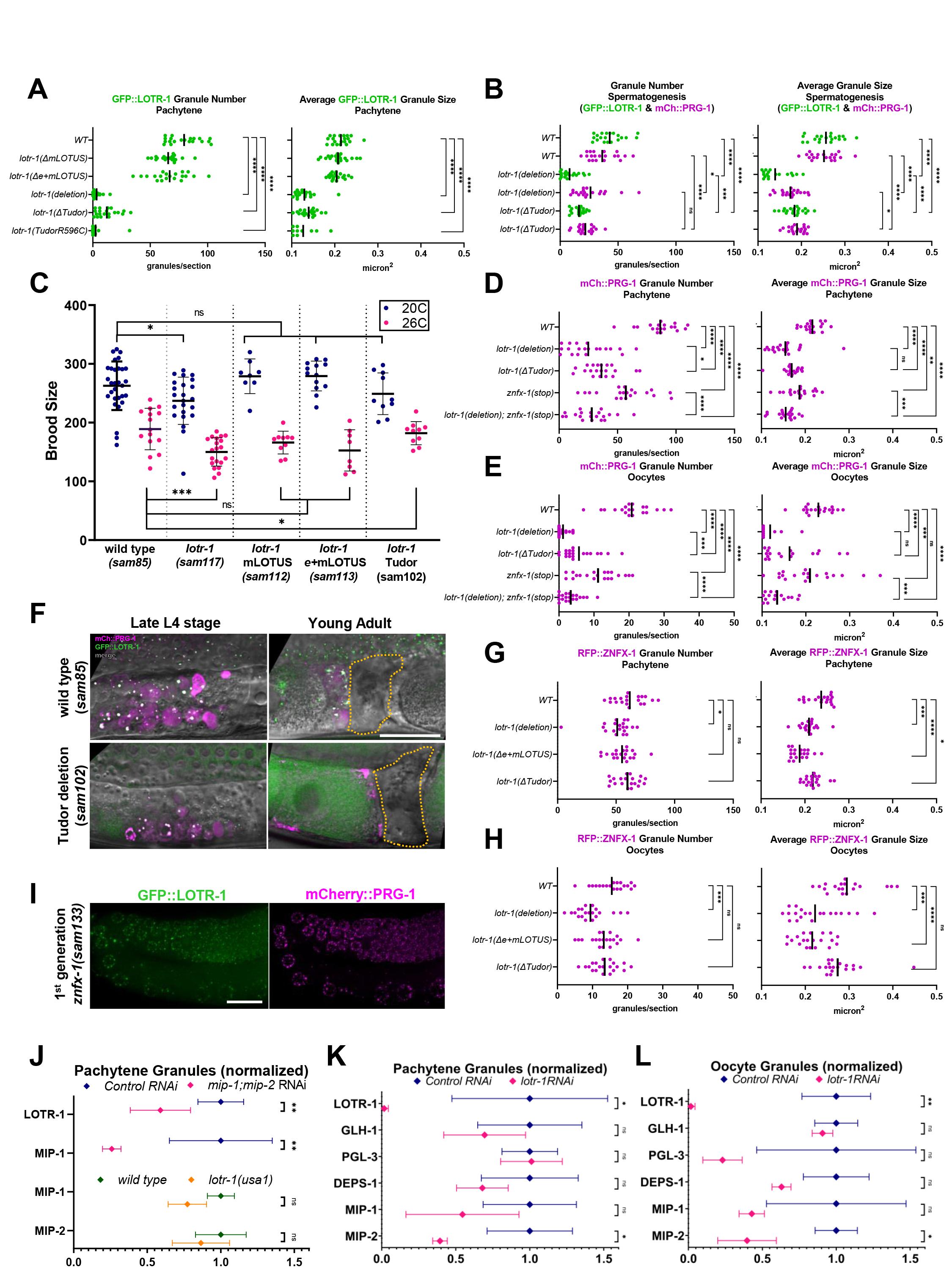

Supplement: S2 Fig — A) Comparison of LOTR-1 granule number and size in the pachytene region of lotr-1 mutants. B) Comparison of LOTR-1 and PRG-1 granule number and size during spermatogenesis in lotr-1 mutants. C) Brood size at permissive (20°C) and restrictive (26°C) temperatures in indicated lotr-1 mutants. PRG-1 granule number and size in D) pachytene and E) oocyte regions of lotr-1 and/ or znfx-1 mutants. F) mCherry::PRG-1 and GFP::LOTR-1 distribution during spermatogenesis in both the presence and absence of LOTR-1’s Tudor domain. ZNFX-1 granule number and size in G) pachytene and H) oocyte regions of lotr-1 mutants. I) Comparison of GFP::LOTR-1 and mCherry::PRG-1 expression in the germlines of first generation znfx-1 mutant worms. J) Normalized granule counts in the pachytene region of GFP-tagged LOTR-1, MIP-1, and MIP-2 granules following empty vector control and mip-1;mip-2 RNAi (top), and in wild type and a lotr-1 deletion allele (bottom). Normalized granule counts in the K) pachytene and L) oocyte regions of GFP-tagged LOTR-1, GLH-1, PGL-3, DEPS-1, MIP-1, and MIP-2 granules following empty vector control and lotr-1 RNAi. P-values from unpaired t-tests, where p>0.05 is ns, p<0.05 is *, p<0.01 is **, p<0.001 is ***, and p<0.0001 is ****. (TIF) [file pgen.1010245.s002.tif]

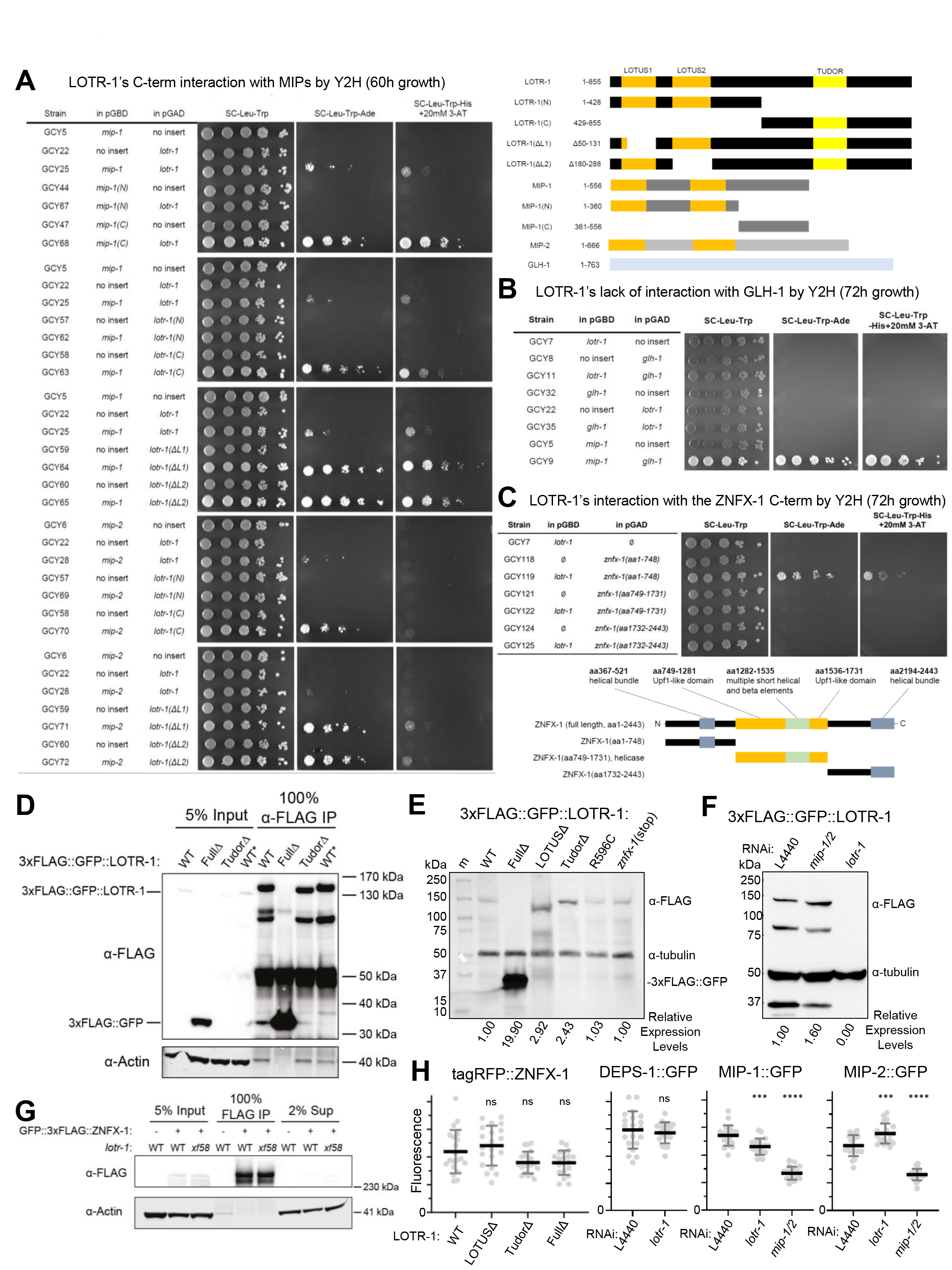

Supplement: S3 Fig — A) Y2H analysis of MIP-1, MIP-2, and LOTR-1. MIP-1’s C-terminal half interacts with full length LOTR-1. LOTR-1’s C-terminal half interacts with both MIP-1 and MIP-2, and the LOTR-1/MIP interactions are independent of LOTR-1’s LOTUS domains. B) Y2H analysis of LOTR-1, MIP-1 and GLH-1 do not uncover an interaction between LOTR-1 and GLH-1. C) Y2H activation through the N-terminal third of ZNFX-1 is strengthened by an association with LOTR-1. D) Western blot for LOTR-1 immunoprecipitations with strains and anti-FLAG antibody used for IP-qMS. E) Western blot showing 3xFLAG::GFP::LOTR-1 expression in mutant backgrounds, α-tubulin loading control. F) Western blot showing 3xFLAG::GFP::LOTR-1 expression following RNAi, α-tubulin loading control. Mean RFP::ZNFX-1 expression intensity in the pachytene region of wild-type and lotr-1 mutants. G) ZNFX-1 immunoprecipitations with the strains and anti-FLAG antibody used for IP-qMS. Done using synchronized young adult worms. Mutations in lotr-1 are indicated above the blots. H) Mean fluorescence intensity of tagged ZNFX-1, DEPS-1, MIP-1 and MIP-2 in the pachytene region of control RNAi, lotr-1 RNAi, and mip-1; mip-2 RNAi. P-values from unpaired t-tests, where p<0.001 is ***, and p<0.0001 is ****. (TIF) [file pgen.1010245.s003.tif]

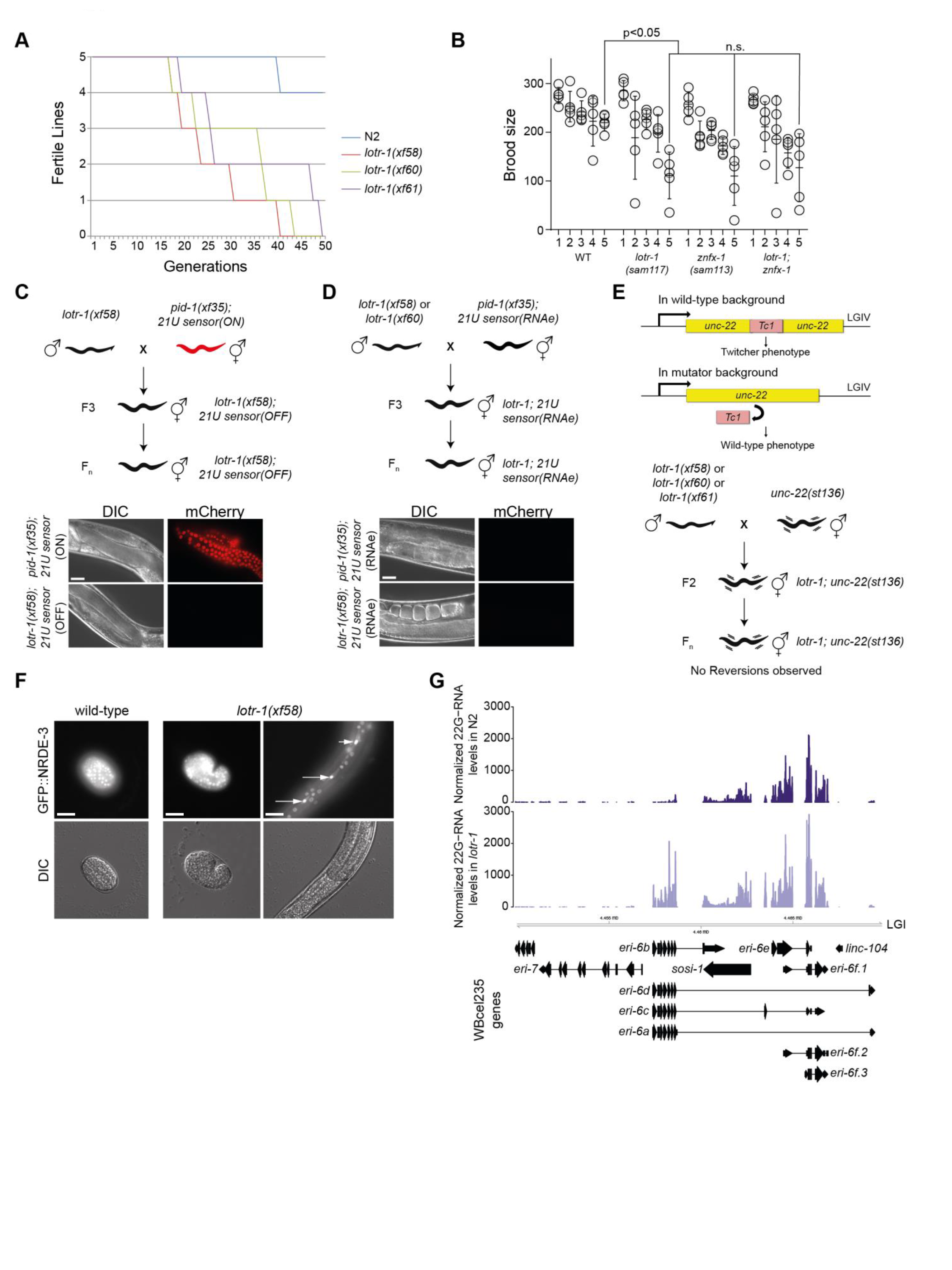

Supplement: S4 Fig — A) Number of fertile lines of each strain indicated per generation. The onset of sterility of lotr-1 mutants occurred at the 18th generation in the xf58 and xf60 alleles, and at the 20th generation in the xf61 allele. The decline in fertility proceeded in all lotr-1 strains until no fertile line remained. B) Brood sizes at 25°C in lotr-1 and znfx-1 mutants over five generations. A T-test was used to determine assess significance at generation five. C) Cross scheme of a non-stably silenced 21U-RNA sensor with lotr-1(xf58) mutants. The F3 of the indicated genotype was scored for mCherry expression in the germline, bottom shows photomicrograph example. D) Schematics of two independent crosses between two lotr-1 mutant alleles and a 21U-RNA sensor that is stably silenced under RNAe, bottom shows representative photomicrograph. E) Schematic of the unc-22(st136) allele and of the crosses performed. In an otherwise wild-type background, the Tc1 copy integrated in the unc-22 gene does not mobilize, and these mutants display a twitcher phenotype. However, if transposon silencing is compromised Tc1 will become mobile and transpose leaving an intact unc-22 gene, which restores the wild-type phenotype. Layout of the unc-22(st136) x lotr-1 crosses to address Tc1 derepression is also shown. No events of phenotypic reversion were found in lotr-1 mutants. F) Differential interference contrast (DIC), and fluorescence photomicrographs of embryos and L4 animals of the indicated genotypes. GFP::NRDE-3 is observed in the nuclei of hypodermic seam cells, indicated by white arrowheads. The images are representative of at least 10 embryos or 10 L4 worms. Scale is 20 microns. G) Genome tracks showing normalized 22G-RNA levels in the eri-6/7 locus of wild type and lotr-1(sam117) animals. (TIF) [file pgen.1010245.s004.tif]

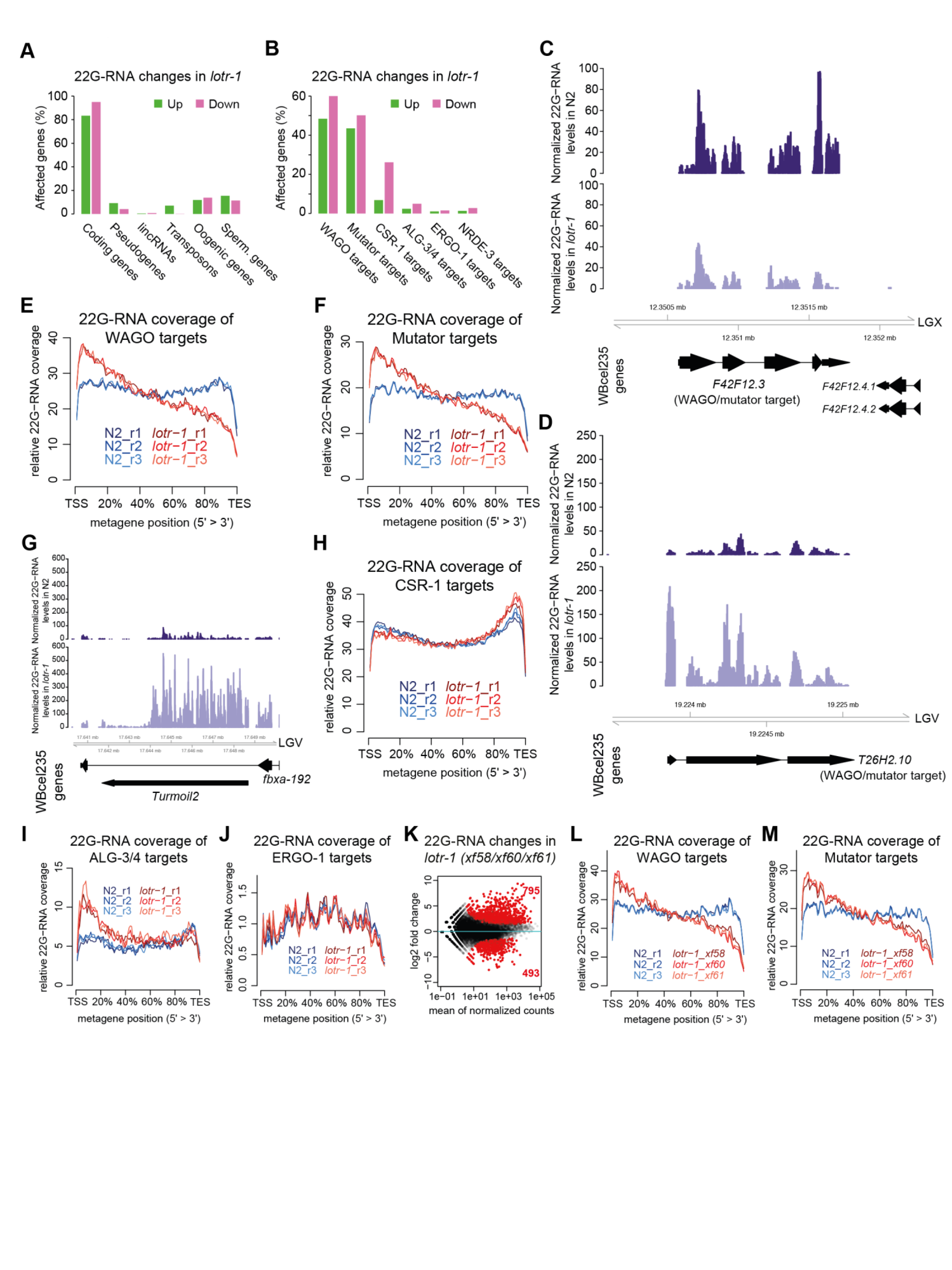

Supplement: S5 Fig — A-B) Bar plots depicting the fraction of genes with up- or down-regulated 22G-RNAs in lotr-1(sam117) mutant belonging to the indicated gene classes (A) and target gene lists (B) defined in previous studies. C-D) Genome tracks of 22G-RNA coverage in wild type (N2) and lotr-1(sam117) mutant depicting two representative genes showing down-regulation (C) and up-regulation (D), as well as 5’ shift in 22G-RNA distribution. E-F,H-J) Metagene plots to visualize the relative 22G-RNA distribution in wild-type (N2) and lotr-1(sam117) mutant over target gene sets of WAGO (E), mutator (F), CSR-1 (H), ALG-3/4 (I) or ERGO-1 (J). TSS/TES, transcription start/end site. G) Genome tracks of 22G-RNA coverage of Turmoil2, a transposable element located within an intron of the gene fbxa-192 targeted by 22G-RNAs, in wild-type (N2), upper track, and lotr-1(sam117) mutant, lower track. K) Differential analysis MA plot of 22G-RNA changes in three different lotr-1 alleles (xf58, xf60, xf61) with partial deletions in the lotr-1 gene. The vast majority (77%) of the deregulated genes highlighted in red color (>2-fold at 10% FDR) are also similarly affected in the lotr-1 (sam117) null mutant shown in Fig 4B. L-M) Relative metagene plots over WAGO and mutator target genes in wild-type (N2) and lotr-1(xf58/xf60/xf61) mutants show very similar profiles to lotr-1(sam117) mutant. (TIF) [file pgen.1010245.s005.tif]
